# MasABK Proteins Interact with Proteins of the Type IV Pilin System to Affect Social Motility of *Myxococcus xanthus*

**DOI:** 10.1371/journal.pone.0054557

**Published:** 2013-01-16

**Authors:** Sarah Fremgen, Amanda Williams, Gou Furusawa, Katarzyna Dziewanowska, Matthew Settles, Patricia Hartzell

**Affiliations:** Department of Biological Sciences, University of Idaho, Moscow, Idaho, United States of America; University of Texas-Houston Medical School, United States of America

## Abstract

Gliding motility is critical for normal development of spore-filled fruiting bodies in the soil bacterium *Myxococcus xanthus*. Mutations in *mgl* block motility and development but one *mgl* allele can be suppressed by a mutation in *masK*, the last gene in an operon adjacent to the *mgl* operon. Deletion of the entire 5.5 kb *masABK* operon crippled gliding and fruiting body development and decreased sporulation. Expression of *pilAGHI*, which encodes type IV pili (TFP) components essential for social (S) gliding, several cryptic *pil* genes, and a LuxR family protein were reduced significantly in the Δ*mas* mutant while expression of the myxalamide operon was increased significantly. Localization and two-hybrid analysis suggest that the three Mas proteins form a membrane complex. MasA-PhoA fusions confirmed that MasA is an integral cytoplasmic membrane protein with a ≈100 amino acid periplasmic domain. Results from yeast two-hybrid assays showed that MasA interacts with the lipoprotein MasB and MasK, a protein kinase and that MasB and MasK interact with one another. Additionally, yeast two-hybrid analysis revealed a physical interaction between two gene products of the *mas* operon, MasA and MasB, and PilA. Deletion of *mas* may be accompanied by compensatory mutations since complementation of the Δ*mas* social gliding and developmental defects required addition of both *pilA* and *masABK*.

## Introduction

The Gram-negative δ-proteobacterium *Myxococcus xanthus* provides a model for social interaction and gliding motility. Gliding motility is defined as movement without the use of flagella and has been well characterized to involve two discrete motility systems in *M. xanthus* [1]. Though there may be crosstalk between the systems in order to coordinate complex behavior such as chemotaxis or predation, each system can function relatively independently of the other. Adventurous (A) motility allows for individual cells to translocate across a substratum while social (S) motility requires cell-cell contact [2].

Social motility generates force by extension and retraction of type IV pili (TFP) and requires that *M. xanthus* cells be separated by less than one cell length [3]. Although S-motility is similar to “twitching” motility in *Neisseria* and *Pseudomonas*, TFP in *M. xanthus* do not adhere to the substratum [4]–[7]. Rather, they adhere to extracellular components such as fibrils and exopolysaccharides on an adjacent cell, which stimulate retraction of the pili [8]. When these signals are absent, cells are non-motile regardless of the integrity of the social motor, which indicates that signaling via cell surface material is inherently crucial to S-motility.

TFP and the required assembly machinery are encoded in *Myxococcus xanthus* in a cluster of 16 genes comprising over 20 kilobases of contiguous sequence. PilA is the structural subunit of pilin in *M. xanthus* and expression of PilA is required for social motility [3]. It has shown to be autoregulatory; however its expression is also dependent on the operon located immediately upstream of the *pil* gene cluster [9]. PilS and PilR are members of a two-component system that regulates the expression of PilA. PilR is a σ^54^-dependent transcriptional activator that binds upstream of *pilA* in *Pseudomonas aeruginosa* [10]. In *M. xanthus*, PilR has been found to be required for expression of *pilA*, while PilS, the sensor histidine kinase, negatively regulates *pilA* expression [9]. Two independent ATPases, PilB and PilT, are responsible for extrusion and retraction of mature pili and both are necessary for gliding motility. PilB has been shown to be responsible for the extrusion of pili from the leading end of the cell, while PilT is responsible for retraction [11], [12]. A deletion in any of the genes encoded in the *pil* cluster is sufficient to disrupt social motility and, in some cases, disrupt or delay development in *M. xanthus*.

One of the proteins involved in crosstalk between the A- and S-motility systems is called MglA. MglA is a Ras-like GTPase that is required for the coordination of both motility motors and a single mutation in this gene is sufficient to abolish swarming, motility and development [13], [14]. The *mglA8* missense mutation produced WT levels of mutant MglA protein, however it failed to properly coordinate motility within *M. xanthus*, leading to an A^−^S^−^ motility phenotype in the mutant with otherwise functional motility systems [13]. *mglA8* (L93F) proved fruitful in the hunt for interaction partners using suppressor mutations [15]. UV and chemical mutagenesis yielded a mutant that recovered partial social motility and carried a second-site mutation that mapped to the third gene of the 5.5 kb operon upstream of the *mgl* operon. This allele-specific suppressor, named *masK* (*mglA*8 suppressor) was predicted to encode a protein kinase [15]. Recombinant MasK in *E. coli* cross-reacted with anti-phosphotyrosine antibody which also reacted with a protein similar in size to MasK in *M. xanthus* that was associated with the inner membrane. MasK was confirmed by yeast two-hybrid assay to be an interacting partner of MglA [15]. The partial restoration of social motility in *mglA8 masK815* mutant coincided with a dramatic increase in the accumulation of FibA detectable by Western blot, the zinc metalloprotease implicated in lipid-directed chemotaxis of *M. xanthus* [16].

In this paper, the phenotype of a deletion of the *mas* operon in *Myxococcus xanthus* was examined. The results show that *masABK* is required for the development of starvation-induced fruiting bodies as well as swarming behavior and gliding. Although swarming on both 1.5 and 0.3% agar was diminished in the mutant, detailed analysis of A- and S- gliding functions and the transcriptome showed that Mas proteins are needed primarily for social gliding. PilA expression and the formation of type IV pili was abolished when *masABK* was deleted. Yeast two-hybrid assays proposed physical interactions between all three members of the *mas* operon, suggesting they form a membrane associated complex, as well as two members of the *mas* operon and the type IV pilin subunit PilA. Interactions between Mas proteins and the pilin provide a possible mechanism for the function of the Mas proteins in the regulation of social behavior in *M. xanthus*.

## Results

### The *masABK* operon encodes three membrane-associated proteins

**Figure 1:**
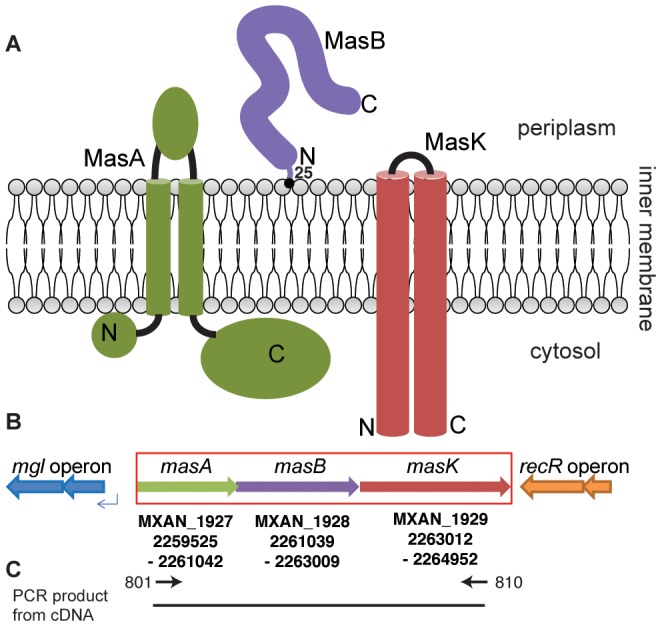
The *mas* operon is predicted to encode three proteins, MXAN_1927 (MasA), MXAN_1928 (MasB) and MXAN_1929 (MasK). A: MasA possesses two transmembrane helices, and a periplasmic domain of 100 amino acid residues (green). MasB is a membrane bound lipoprotein (purple), anchored to the inner membrane at residue 25 (black dot). MasK is a membrane-associated protein kinase (red) [15]. B: Genomic context of the *masABK* operon. The *mas* operon is 327 bp upstream of the *mglBA* operon (blue arrows) and is transcribed from the opposite strand. The *mas* operon coordinates are indicated below the ORFs for reference. The spacing of the three predicted open reading frames (ORF) suggests that the genes are cotranscribed. C: Primer extension analysis of cDNA transcript. cDNA transcript was generated using a primer specific for MasK, 810, and the template was amplified using primers 801 and 810 (illustrated using inward arrows). The resulting amplicon was 5.4 kb and confirms expression of the *masABK* operon involves the formation of a polycistronic mRNA transcript.

*masK* is the third gene of an operon upstream of and divergent with *mglBA*. Upstream of *masK* are two open reading frames which have been named *masA* and *masB* with predicted protein sizes of 505 and 656 amino acids, respectively. *masA* and *masB* have an overlap of 3 bp, while *masB* and *masK* have an intergenic gap of 3 bp. Fig. 1 depicts both the predicted localization and membrane topology of the three members of the *masABK* operon as well as the genomic context of the *mas* operon. The *mgl* operon (indicated as blue arrows in Fig. 1B) is located 327 bp upstream of *masA* on the complimentary strand, while the downstream operon including a *recR* gene (orange, Fig. 1B) terminates 37 bp downstream of the stop codon of *masK* on the complimentary strand.

In order to determine if these three open reading frames are cotranscribed as part of an operon, mRNA was isolated from the WT strain. After treatment with DNaseI to remove any remaining genomic DNA, cDNA was generated using a random hexanucleotide primer. As shown in Fig. 1C, amplification of cDNA using primers 801 and 810 yielded a ≈ 5.4 k bp product (predicted size 5444 bp) that was absent in a Δ*masABK* cDNA template and genomic WT template that was DNaseI digested (data not shown). This demonstrates that *masA, masB and masK* are cotranscribed in one polycistronic mRNA transcript.

**Figure 2:**
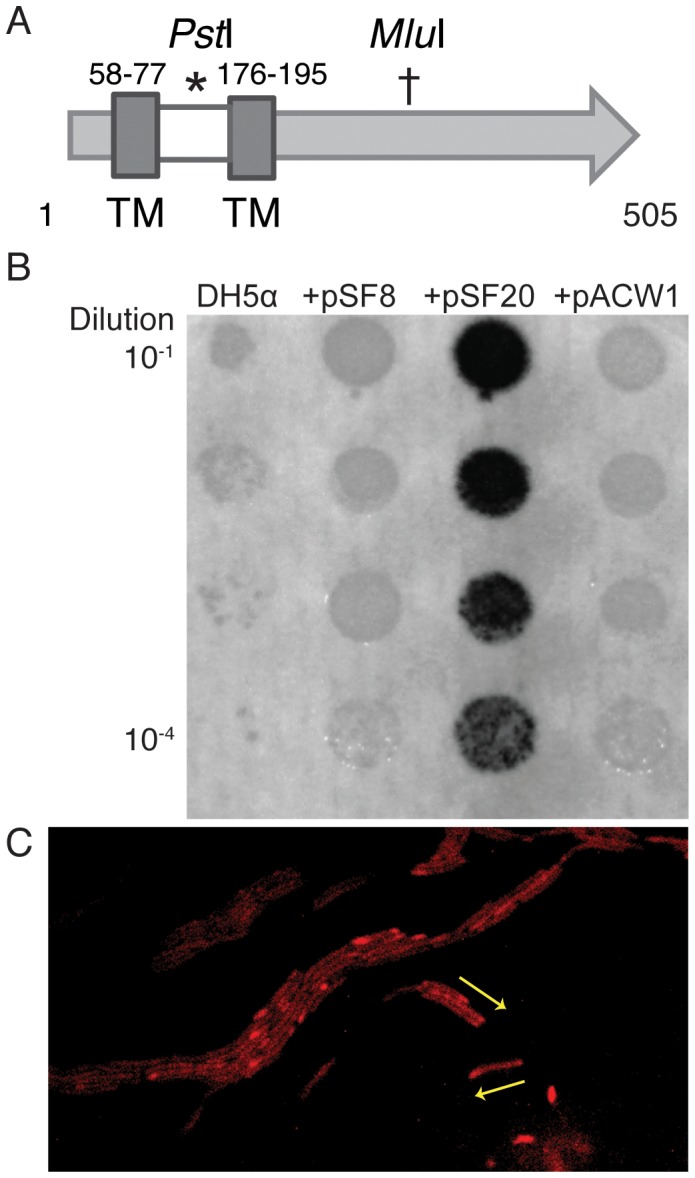
MasA and MasK are membrane proteins. A. MasA has two predicted membrane spanning regions (TM  =  transmembrane). B. PhoA fusions demonstrate that MasA has a periplasmic domain. MasA was cloned into pCR Blunt II TOPO under a *lac* promoter to form pSF8. pSF20 is a derivative of pSF8 with an in-frame insertion of the *E. coli phoA* gene at the *Pst*I site (*), while pACW1 has an in-frame fusion at the *Mlu*I site (†) shown in panel A. Overnight cultures were adjusted to an OD_600_ of 1.0 and serially diluted. 5 μl of each dilution was incubated on LB agar with 35 µg/ml X-P at 37°C for 16 hours. C. Fluorescence from a MasK-mcherry fusion expressed in an otherwise WT strain was concentrated at one pole of the cell. Yellow arrows indicate the direction of movement of cells on 1.5% agar surface.

*masA* (MXAN_1927) is annotated as a hypothetical protein of 55 kDa, while *masB* (MXAN_1928) is annotated as a putative lipoprotein of predicted mass 68 kDa [17], [18]. Since MasA has no significant homology to any characterized proteins, information about the characteristics of the protein was sought. Two transmembrane regions (residues 58 to 77 and 177 to 195) in MasA were predicted using TMPred [19], however HMMTop [20], [21] and PRED-TMR [22] both identified only the region from 58 to 77 as a transmembrane region. If the former prediction is true, MasA should reside in the inner membrane with a 100 amino acid periplasmic domain; however, if the latter is the case, MasA should reside in the periplasm from residue 1 to 176, and the cytoplasm from residue 196 to 505. The schematic in Fig. 2A illustrates both TMPred-predicted transmembrane domains as well as the putative periplasmic region.

To examine this prediction, two in-frame translational fusions with MasA were constructed using the gene encoding the *E. coli* alkaline phosphatase (*phoA*). One fusion was located in the predicted periplasmic domain as indicated by the asterisk (*) visible in Fig. 2A, while the other was in the predicted cytoplasmic region indicated by the (†). Alkaline phosphatase hydrolyzes the colorless reagent 5-bromo-4-chloro-3-indolyl phosphate (BCIP, X-phosphate, XP) into inorganic phosphate and 5-bromo-4-chloro-3-indole, which produces an insoluble blue pigment. As PhoA is only active when exposed in the periplasm, indigo coloration in a colony is indicative of periplasmic location of this protein fusion [23], [24]. When we expressed a PhoA fusion (pSF20) that was present at the *Pst*I site, blue pigment accumulated, indicating a periplasmic location of PhoA as shown in Fig. 2B. However, when we expressed the fusion at the *Mlu*I site (pACW1), no dye was observed, leading us to conclude that the fusion at this position was inactive or possibly unstable. These results support the TMPred-predicted topology for MasA as an integral membrane protein with a periplasmic domain at residues 78 to 176, with two transmembrane regions.

The product of the third gene in the operon, MasK, was previously shown to be associated with the inner membrane fraction of *M. xanthus* [15] and despite the fact that MasK lacks an apparent signal sequence, PSORTb 3.0 [25] places it in the cytoplasmic membrane and TMPRED predicts two transmembrane helices. To determine the location of MasK, a plasmid bearing *masK-mcherry* was integrated into the WT strain. Fluorescence images of strain MxH2639 showed fluorescence from MasK-mcherry that was more focused at one cell pole. Although the fluorescence signal was insufficient to capture video images, comparison of still images with visual tracking of cells on agar pads showed that in 11 of 15 cells that were moving, the most intense fluorescent spot was at the leading pole of the cell (Fig. 2C). Although fluorescence can observed elsewhere in the cell, many motility proteins including the MasK interacting partner MglA have been shown to oscillate between poles during periods surrounding reversals, and it is possible that the fluorescence is not due to background but the dynamic localization of MasK [26]–[28].

### Deletion of *masABK* abolishs social motility

To understand the role of the *mas* gene products, *mas* mutants were constructed. As initial attempts to create a disruption of MasK using an internal fragment were unsuccessful, leading to the conclusion that MasK might be essential for vegetative growth [15], the entire operon was deleted from the chromosome. The region deleted in strain MxH2604 (Δ*masABK*) is marked in Fig. 1B with a red box surrounding the *mas* operon. A 1.7 kb region corresponding to the *mglBA* operon and the intervening sequence between the 5′ end of *mglB* and the 5′end of *masA* were cloned into the pBJ114 vector (Kan^R^, *galK* [29]) adjacent to a 1.3 kb region beginning after the *masK* stop codon, including the operon including *recR*. The resulting plasmid, pGF94, was electroporated into the WT *M. xanthus* strain.

Integration of pGF94 into the WT chromosome yielded a merodiploid (MxH2545) with no motility defects (data not shown) that was resistant to kanamycin. After counterselection on medium with galactose, the phenotypes of Kan^s^ colonies, identified by replica plating, were examined. Kan^s^ gal^R^ colonies (n = 2000) displayed two distinct morphologies. One group (n = 1143) produced yellow colonies with WT motility; PCR of these derivatives showed that *mas* was intact as the integrated plasmid pGF94 had undergone excision from the genome. The second group produced yellow colonies that showed reduced motility relative to the WT when plated on 1.5% agar; the *mas* operon was not detected in PCR of these derivatives; instead a fragment corresponding to the deletion (*mglBA* joined to *recR*) confirmed that these strains carried the *mas* operon deletion (Fig. S1). Derivatives displaying the reduced motility phenotype were obtained from three independent counterselection experiments. The Δ*masABK* mutant will be referred to as Δ*mas*.

Although Δ*mas* was designed to minimize perturbations on the products of the *mgl* operon, the possibility that alterations in MglA expression due to polar effects on the *mgl* promoter were responsible for the loss of motility could not be automatically discounted. To confirm that this was not the case, whole cell extracts of WT, Δ*mgl* and Δ*mas* were separated by SDS-PAGE and transferred to a PVDF membrane, which was probed with anti-MglA antibody (1∶1000 dilution). As expected, the WT possessed robust levels of MglA in whole cell extracts, while the corresponding band was absent in the Δ*mgl* strain. No detrimental effect on MglA production was observed when protein extracts were compared between the WT and the Δ*mas* isolates (Fig. S2) which led to the conclusion that loss of MglA due to a deletion of a *cis*-acting element was not responsible for the motility defect in a Δ*mas* strain.

The small colony size of the Δ*mas* mutants might be due to a defect in motility or growth. When Δ*mas* mutants were cultured in rich (CTPM) medium alongside the WT parent, no vegetative growth defect was observed. A range of assays was conducted to determine if small colony size of Δ*mas* was due to specific defects in the motility system. These include examination of colony edge morphology to look for evidence of groups of cells (S-swarms) and isolated cells (A-swarms), assays to quantify swarm expansion on sift and hard surfaces, and microscopic assays to quantify the ability of single cells to move, the gliding rate and the frequency of cell reversals. Finally a genetic test was used to determine if the *masABK* genes are central to the gliding systems.

**Figure 3:**
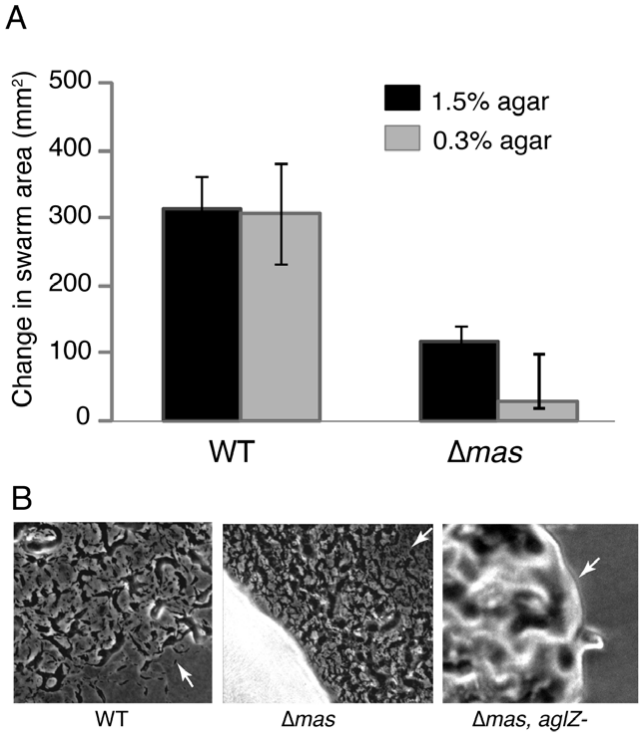
Deletion of the *masABK* operon affects swarming. A: WT and the Δ*mas* strain were grown in CTPM to a concentration of 5×10^8^ cells/ml. After 10-fold concentration, 3 µl were plated on 1.5% and 0.3% agar plates as previously described [1]. Swarm diameters was measured after 96 hours. Data represent an average of nine samples plated in three separate assays. B: Photomicrographs of the colony edge after 4 days on CTPM 1.5% agar reveal isolated cells indicative of A-motility. White arrows designate the leading edge of the colony.

As shown in Fig. 3A, deletion of the *masABK* operon resulted in a >90% decrease on 0.3% (soft) agar and a 70% decrease in swarming on a 1.5% (hard) agar surface compared to the WT. Decreased swarming on soft agar is usually associated with defects in S-gliding whereas decreased swarming on hard agar is usually associated with defects in A-gliding. This is consistent with previous observations that the suppressing mutation in *masK* restored social motility to an otherwise A^−^S^−^ strain [15]. Moreover, the small, heaped colony of the Δ*mas* mutant resembles that of the Δ*mglBA* colony but in contrast, A-gliding cells are present at the edge of the Δ*mas* mutant as shown in Fig. 3B. This indicated that the A-gliding system is functional in the Δ*mas* mutant. Swarm expansion measures the ability of cells at high density to glide over different surfaces. Hence the *mas* operon is crucial for swarming at high cell density associated with S and A gliding. As swarms are by their nature social, it is impossible to discount the contribution social motility may make to a swarming assay even on conditions that would otherwise favor A-motility, and swarming is not a sole and comprehensive descriptor of motility defect in *M. xanthus*.

A more refined test of motility involves analysis of cells by time-lapse videomicroscopy. The gliding rate of the Δ*mas* mutant on hard agar was 1.9 µm/min, which does not differ significantly from the rate of 2.3 µm/min obtained for the WT. The gliding rate and the presence of isolated cells at the colony edge show that deleting *mas* does not affect A-gliding per se. Additionally, the frequency of cell reversals was similar in the WT and the mutant. S-gliding motility in *M. xanthus* requires cell-cell contact in order to function, however this proximity causes difficulty in calculating individual cell velocities using tracking software because the cells are not well isolated. To get around this problem, social motility of single cells is quantified using the chemical methylcellulose, which has been shown to stimulate retraction of type IV pili in *M. xanthus* in the absence of cell contact [8]. When WT *M. xanthus* cells were assayed in a 0.5% methylcellulose solution, the average velocity was 10–12 µm/min (n = 50 cells) and under the same conditions, Δ*mas* mutant failed to glide (<1.0 µm/min, n = 50 cells).

Disruption of the *aglZ* gene is sufficient to abolish A-motility in an otherwise WT strain. When pAGS164, a plasmid shown to disrupt the *aglZ* gene [30], was introduced into Δ*mas*, it too abolished single cell motility at the colony edge Fig. 3B, third panel. A second mutation in a known A-motility gene such as *aglZ* renders Δ*mas* strains completely non-motile at the colony edge, which confirms that *masABK* function primarily in S-motility.

Dye-binding and cohesion assays were performed to quantify changes to the extracellular matrix (fibrils or EPS). Both trypan blue and Congo red dyes can identify differences in the production of EPS [31]. Both of the dye assays showed that the Δ*mas* mutant produces less EPS because Congo red binding in Δ*mas* was 73.8% that of the WT as shown in Fig. S3. The cohesion (aka aggregation) assay provides an independent measure of cell surface components. Under the conditions of this assay, WT cells aggregate and fall out of solution. After thirty minutes, the cell density (Klett reading) of WT samples was reduced from 100 to ≈50 and dropped to less than 10 by 3 hours. By comparison, the *mas* strain required 3 h to decrease from 100 to 50 Klett units and another 21 h to reach a Klett of 10. Taken together, these data show that although EPS production is reduced in the Δ*mas* mutant, the reduction is modest compared to the *pilA* mutant [32], [33]. This suggests that Δ*mas* can partially suppress the loss of PilA with regard to changes in the extracellular matrix.

### Deletion of *masABK* affects expression of certain *pil* genes, a LuxR family protein and myxalamide

The Δ*mas* motility phenotype is very similar to a well-characterized S^−^ mutant, Δ*pilA* (DK10407). *pilA* encodes the structural subunit of type IV pili which is necessary for social motility [3]. When compared alongside the Δ*mas* mutant, we found that Δ*pilA* also swarmed poorly on hard agar (70% decrease relative to WT) and failed to swarm on soft agar (>90% decrease relative to the WT). To determine how deletion of *mas* impacted motility, we first asked if transcription of known gliding genes was altered in the Δ*mas* mutant. To this end, a DNA microarray was performed to assay global changes in the mRNA transcriptome.

The array results, summarized in Table 1, represent the statistically significant changes compiled from nine datasets (three independent biological and technical assays) that represent genes that were unique to the Δ*mas* mutant. Significantly, expression of TFP-associated genes of two operons in *M. xanthus* MXAN_0360–0364 and MXAN_5780–5783 was decreased 23 to74-fold in the Δ*mas* mutant compared to the WT. The short (≈3 bp) overlaps between genes MXAN_0360–0364 suggest that this is an operon. MXAN_0360, 0361 and 0362 are predicted to encode pilin biogenesis proteins, yet they are not known to function in *M. xanthus* gliding activity. The N-terminal region of MXAN_0360 is similar to PilW and is in the PulG family (COG2165), hinting at a role in pilin biogenesis or secretion. MXAN_0361 is similar to PilV and also falls in the PulG family. MXAN_0362 is in the *Neisseria* PilC superfamily, which contains a ß-propeller domain and has a tip-associated adhesion PilY1 domain. MXAN_0363 shares some identity (36/117) with *Sphingomonas* cytochrome C. MXAN_0364, annotated as a hypothetical gene, shows 38% identity and 56% similiarity over 64 residues to PilX, which functions in protein secretion of fimbriae in *P. aeruginosa* [34]. Genes MXAN_5780–5783 form a single operon encoding PilI, PilH, PilG and PilA respectively and are essential for S-motility in *M. xanthus*. Expression of all four genes in the *pilA* operon was decreased significantly, whereas expression of nearby *pil* genes was unchanged. PilA is the major pilin subunit while PilG, PilH and PilI are members of an ABC-exporter complex regulated by the same promoter as *pilA* [35].

**Table 1:** Genes with altered expression in Δ*mas* relative to the WT.

Gene locus	Gene function/annotation	Fold decrease
	**Pili and associated biogenesis proteins**	
MXAN_0360	type 4 pilus biogenesis operon protein	24
MXAN_0361	type 4 pilus biogenesis operon protein	46
MXAN_0362	putative pilus biogenesis operon protein	23
MXAN_0363	hypothetical protein	28
MXAN_0364	hypothetical protein	26
MXAN_5780	putative efflux ABC transporter, permease protein *pilI*	23
MXAN_5781	efflux ABC transporter, ATP-binding protein *pilH*	26
MXAN_5782	putative efflux ABC transporter accessory factor *pilG*	44
MXAN_5783	pilin (*pilA*)	74
	**Signaling components**	
MXAN_2230	transcriptional regulator, LuxR family	196
MXAN_3605	response regulator	28
MXAN_3606	putative sensor histidine kinase	52
	**Other: including putative lipoproteins, protease**	
MXAN_0647	putative lipoprotein	34
MXAN_7000	putative lipoprotein	46
MXAN_2221	peptidase, M27 (PrtB) family	26
MXAN_5478	LysM domain protein	48
MXAN_7017	CRISPR-associated protein, Csd3 family (*csd3*)	23
	**Proteins of unknown function/target**	
MXAN_1904	conserved hypothetical protein	85
MXAN_3175	hypothetical protein	109
MXAN_4536	hypothetical protein	60
MXAN_4538	hypothetical protein	34
MXAN_5039	conserved hypothetical protein	64
MXAN_5040	aldehyde dehydrogenase family protein	45
MXAN_6798	conserved hypothetical protein	66
MXAN_4123	hypothetical protein	23

Gene locus	Gene function/annotation	Fold increase
	**Myxalamide gene cluster**	
MXAN_4526	polyketide synthase type I	13
MXAN_4527	polyketide synthase	12
MXAN_4528	polyketide synthase	12
MXAN_4529	polyketide synthase type I	16
MXAN_4530	polyketide synthase type I	29
	**Misc: putative lipoproteins, nucleases, protease, enzymes**	
MXAN_3352	pseudouridine synthase, RluA family	14
MXAN_5728	ribonuclease H (*rnhA*)	21
MXAN_7511	ribonuclease P protein component (*rnpA*)	20
MXAN_0442	putative lipoprotein	14
MXAN_4540	putative lipoprotein	25
MXAN_2404	FKBP-type peptidyl-prolyl cis-trans isomerase SlyD (*slyD*)	17
MXAN_6913	cytochrome d ubiquinol oxidase, subunit I (*cydA*)	16
	**Hypothetical proteins/proteins of unknown function**	
MXAN_0999	hypothetical protein	14
MXAN_1165	hypothetical protein	18
MXAN_5129	hypothetical protein	13
MXAN_7510	conserved hypothetical protein TIGR00278	20

Genes that encode regulators or proteins that may participate in synthesis of signals were decreased significantly in the mutant. Expression of MXAN_2230, which encodes LuxR-type transcriptional regulator was down almost 200-fold. The N-terminal region (residues 50–175) of MXAN_2230 contains a GAF-2 domain (pfam3185) while the C-terminal region (≈residues 275–360) contain CitB and LuxR C-like HTH superfamily (cd06170) domains that include residues involved in DNA binding and dimerization. This GAF domain is present in phytochromes and cGMP-specific phosphodiesterases. An uncharacterized two-component system (TCS) at MXAN_3605 and 3606 (response regulator and histidine kinase, respectively) exhibited diminished expression in the Δ*mas* strain.

Two putative lipoproteins showed decreased expression in a Δ*mas* strain relative to the WT, MXAN_0647 and 7000. Changes in the lipoproteins may reflect alterations in the membranes or extracellular environment. Two proteolytic enzymes, such as MXAN_2221, which encodes a member of the *prtB* family of proteases, were found to have decreased expression in a Δ*mas* strain. MXAN_5478 is a LysM domain protein, and showed a 48-fold lower expression in the Δ*mas* strain. LysM-domains are occasionally involved in binding peptidoglycan for enzymatic degradation of cell wall components [36]. Expression of MXAN_7017, which encodes a CRISPR-associated protein of the Csd3 family, was decreased. CRISPR sequences in a bacterial genome provide resistance against viruses and other mobile DNA elements, which may mean that a decrease in the expression of bacterial resistance may make this strain more susceptible to viral infection or other DNA integrations [37]. Several genes encoding hypothetical proteins (MXAN_1904, MXAN_3175, MXAN_4536, MXAN 5039 and 5040) showed a decreased transcript level in the Δ*mas* strain.

Decreased expression of a hypothetical protein encoded by MXAN_4123 may provide partial insight to the developmental defect of the Δ*mas* mutant because expression of this gene is normally increased during development (K. Dziewanowska and P. Hartzell, unpublished results).

Genes with increased expression in a Δ*mas* strain include five loci encoding polyketide synthases. As shown in Table 1, the cluster of MXAN_4526 to MXAN_4530 demonstrated an increase in RNA expression between 12 and 29-fold relative to the WT. The gene cluster from MXAN_4526 to MXAN_4530 has been identified as the one responsible for the synthesis of myxalamide, an inhibitor of electron transport in eukaryotes [38].

Genes predicted to be involved in the modification or degradation of RNA *in vivo* also displayed increased expression. MXAN_3352, which encodes a pseudouridine synthase of the RluA family and genes encoding ribonucleases H and P, had increased levels of expression in a Δ*mas* strain. Two genes encoding putative lipoproteins, MXAN_0442 and 4540, had increased transcript levels suggesting that there may be altered cell-surface secretion in a Δ*mas* strain, leading to changes in signaling or motility. Additionally, protein modification may be affected as expression of MXAN_2404, which encodes an FKBP-type peptidyl-prolyl *cis-trans* isomerase SlyD was increased. SlyD protein is predicted to act as a chaperone. Several hypothetical proteins, MXAN_0999, MXAN_1165, MXAN_5129 and MXAN_7510, showed increased expression relative to the WT. Although addition of *masABK* to MxH2604 (*mas*) was predicted to restore expression of these genes to WT levels, motility was not restored, nor was the expression of any of the genes listed in Table 1 returned to WT levels.

**Figure 4:**
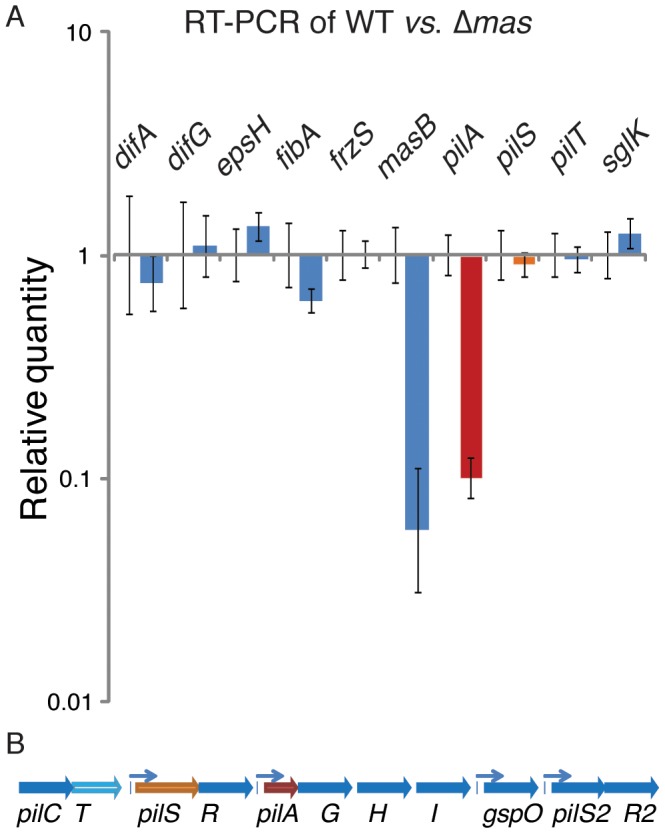
qRT-PCR confirms *pilA* expression decline is specific. A: Relative quantity of S-motility gene expression in *M xanthus* WT and Δ*mas* strains. Gene expression in the Δ*mas* strain was normalized to the WT reference (set to 1) and relative quantitation are represented as a ratio of strain/WT, such as Δ*mas*/WT. Values represent the average of three PCR reactions. *pilA* is shown in red (panels A and B), *pilS* is shown in orange and *pilT* is in lighter blue. B: Genomic context of the *pilA* gene within the 20 kb *pil* gene cluster. Arrows denote known promoter regions for *pilSR, pilAGHI*, *gspO* and *pilS2/R2* transcription units [12], [57].

Results of qRT-PCR confirmed that changes in gene expression in Δ*mas* were specific to a subset of S-motility genes. Although most genes did not show a quantifiable difference in transcription, the TFP subunit *pilA* showed decreased expression (Fig. 4A, red bar). A similar decrease in transcription was observed for *masB*, the deletion control (Fig. 4A, blue bar). Although other genes in the *pil* gene cluster such as *pilS* and *pilT* did not differ significantly in expression from the WT levels, these genes are expressed under the control of different promoters as shown in Fig. 4B. *pilS*, shown in Fig. 4B as an orange arrow, encodes a sensor histidine kinase in a two component system (TCS) with its corresponding transcriptional activator response regulator *pilR* [5]. *pilT* (Fig. 4B, light blue arrow) encodes an ATP-binding cassette protein that is essential for retraction of pili in *M. xanthus* [11], [12]. Neither *pilS* nor *pilT* was expressed in Δ*masABK* at a significantly different level from that observed in the wild-type, which suggests that global regulation of the *pil* gene cluster is not responsible for the defect in social motility observed in the Δ*masABK* strain. *fibA* expression in a Δ*masABK* strain was found to be slightly decreased relative to the WT. FibA was produced in greater quantity in the gain of function mutation masK* (*mas815*; strain MxH1104) [15]. Hence it was not surprising to find a decrease in *fibA* due to loss of function *masABK* mutant.

Significantly, loss of the *mas* operon did not have an effect on other S-motility genes such as *eps*, *sglK*, or *frzS. epsH* encodes a glycosyltransferase essential for exopolysaccharide production [39]. *difA* is a methyl-accepting chemotaxis protein (MCP) required for fibril formation and *difG* is a fibril biogenesis regulator [31], [40]. *frzS* encodes a coiled-coil S-motility protein that interacts with MglA *in vitro* [41]. *sglK* encodes a heat-shock protein chaperone with similarity to DnaK that is critical for development but activated by starvation rather than heat [42]. Therefore, the defect in S-motility from the Δ*mas* mutation appears to be restricted to the pilin gene cluster rather than a global effect on all social motility regulators or signaling systems.

### Loss of *pilA* does not affect expression of *mas*

**Figure 5:**
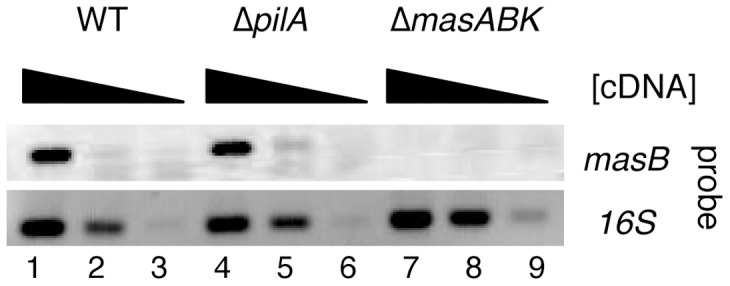
Deletion of *pilA* does not affect *masB* transcription. RNA was harvested from 1×10^8^ cells and mRNA was used to generate cDNA using a random hexanucleotide primer. Gene specific primers for *masB* and the *16S* gene (endogenous control) were used in the amplification reaction with cDNA. *masB* expression was amplified from cDNA using 50, 5 and 0.5 ng template, while *16 S* expression was amplified using 200, 20 and 2 pg cDNA. Both the WT and *pilA* strain express *masB* (black band in lanes 1 and 4 respectively). Hence, deletion of *pilA* does not adversely affect the expression of *masB* in *M. xanthus*. Deletion of *masABK* abolished *masB* expression (lanes 7–9).

As deletion of *mas* adversely affected *pilA* expression in the microarray and RT-PCR, we examined the possibility that deleting *pilA* might cause a similar defect in *mas* expression at the RNA transcript level. Using *masB* as proxy for *masABK*, total mRNA was diluted and probed with gene specific primers to determine whether *mas* expression was affected by the deletion of *pilA*. Resulting amplicons were electrophoresed on an agarose gel to allow rapid identification of strains lacking *masB* transcript. As shown in Fig. 5, a visible product corresponding to *masB* was present in the WT strain (lane 1) but was absent in a Δ*mas* strain, which lacks *masB* (lane 7). When we examined a Δ*pilA* strain, we found no significant difference in the expression of *masB* relative to the WT (lane 4). This suggests that the effect of Δ*mas* on *pilA* expression is unidirectional rather than a bidirectional regulation, meaning that *mas* is still actively transcribed in a *pilA* mutant.

### Addition of *masABK* is not sufficient to restore motility

**Figure 6:**
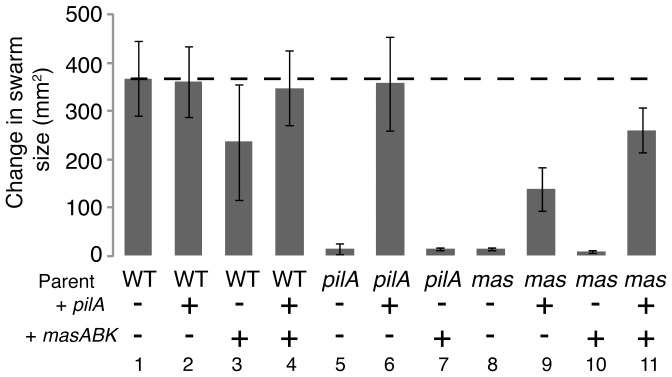
Addition of *pilA* and *masABK* restores swarming to the Δ*mas* mutant. Strains were grown to a density of 5×10^8^ cells per ml and concentrated 10-fold. Aliquots (3 µl) were spotted on 0.3% agar and incubated at 32°C for 96 hr. Bars represent the average of 3 assays with 3 replicates each. Error bars represent the standard deviation. The dashed line (− −) is included for reference and represents the WT average.

In an attempt to complement the Δ*mas* motility defect, a plasmid with a 6.2 kb fragment including the *mas* operon and approximately 600 bp upstream of the start of *masA* (pSF25) was integrated at the native chromosomal site by homologous recombination. The resulting strain yielded kanamycin resistant colonies but failed to restore social motility. mRNA was harvested from this particular strain and analyzed for global gene expression using a cDNA microarray. Similarly, *pilA* expression was neither restored nor was the developmental defect repaired. As integration of pSF25 might adversely affect expression of the *mgl* operon from the promoter present in this particular fragment, we also introduced the *masABK* operon ectopically at the Mx8 phage attachment site *attB* in pSF26. As shown in Fig. 6, addition of *masABK* alone at the chromosomal or *att*-*int* site was not sufficient to restore swarming (column 10).

The inability of *masABK* to restore full motility might be due to a mutation in the reintroduced *masABK*, an incomplete regulatory region, or a pre-existing secondary mutation in the genome. To ensure this failure was not due to a mutation in *masABK*, the insert in plasmid pSF26 was sequenced and found to match the WT genomic sequence. Addition of DNA up to 400 bp upstream of *mas*, which included part of the *mgl* operon, failed to rescue the phenotype. It was considered that a secondary mutation may have arisen during each of the three independent constructions of Δ*masABK*. Given the consistent loss of S-motility and dramatic change in *pilA* expression, a 1.6 kb fragment of the *mas* mutant genome that includes a region necessary and sufficient to complement a Δ*pilA* strain [9] was cloned and sequenced. No point mutations or inverted sequences were detected after sequencing three Δ*mas* isolates. Each showed perfect (1627 bp/1627 bp) identity to the NCBI genomic sequence for *M. xanthus* DK1622, minimizing the likelihood that mutations in the *pilA* gene or regulatory region are causing the phenotype.

As it had previously been demonstrated that S-motility is dependent on a threshold level of *pilA* expression [43], a second WT copy of *pilA* was added in order to determine if motility could be restored. Once the additional copy of *pilA* was integrated at the chromosomal site in a Δ*mas* parent, swarming on soft agar increased from 4% to 38% the WT level. Moreover, addition of the *masABK* operon at the *att* site (pSF26) to a strain already containing pSF34 (WT *pilA*), restored swarming on soft agar to 70% the WT level as shown in Fig. 6. Swarming on hard agar was restored as was motility as the single cell level. As shown in Table 2, addition of both *masABK* and *pilA* complemented the Δ*mas* motility defect and restored nearly WT velocity (10.6±4.8 µm/min) and reversals (20.4 min) but Δ*mas* cells that received only *masABK* failed to glide.). Addition of a second copy of *pilA* to the Δ*masABK* strain increased the velocity (to 4.1±2.1 µm/min) and reversals (11.2 min). *pilA* and Δ*mas* controls were nonmotile. The WT controls translocated at 9.5 µm/min in 0.5% methylcellulose (standard deviation of ±2.7 µm/min, n = 50 cells); cells reversed once every 16.4 min.

**Table 2:** *pilA* and *masABK* are required for motility in 0.5% methylcellulose.

+Strain	Velocity in μm/min	Min/Rev
WT	9.5	16.4
Δ*pilA*	1.2	ND
Δ*mas*	0.7	ND
Δ*mas*+ *pilA*	4.1	11.2
Δ*mas*+*mas*	0.8	ND
Δ*mas*+*pilA*+ *mas*	10.3	20.4

Cells were grown overnight in CTPM with appropriate antibiotics and overlaid with methylcellulose to a final concentration of 0.5% w/v. Velocities were determined using Metamorph (n = 50). Cells above a threshold velocity of 1.5 µm/min were used to calculate the reversal frequencies.

**Figure 7:**
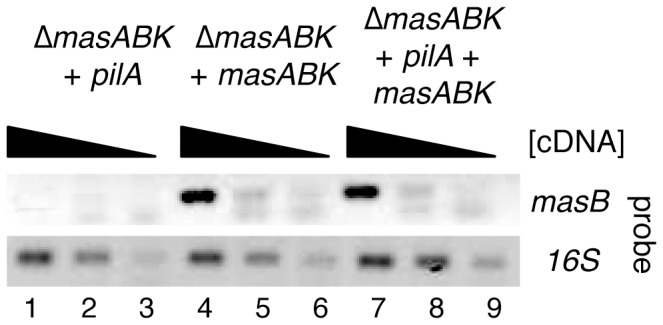
Expression of *masB* is observed from the *masABK* pSF26 construct *in vivo*. See Figure (sqRT-PCR) for RT-PCR conditions. 5 μl of reaction was electrophoresed through TAE agarose. The *masB* transcript is detectible in strains that carry the *masABK* complementing construct (lanes 4 and 7), but absent in lane 1, a strain that lacks *masABK* at either the chromosomal or ectopic site.

In order to confirm that both *masABK* and *pilA* were being expressed in cells that showed some S-swarming recovery, mRNA transcript levels of *masB* as well as accumulation of PilA protein in whole cell extract were examined from all complementing strains as previously described. As shown in Fig. 7, lane 1, when *pilA* was added, no *masB* transcript was detectable. Conversely, addition of *masABK* at the *att* site did restore expression of *masB* gene product independent of the addition of *pilA* (band in Fig. 7, lanes 4 and 7).

**Figure 8:**
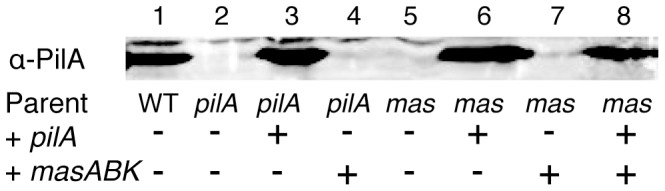
Immunoblot analysis shows that expression of PilA is abolished in a *mas* deletion strain and is only rescued by addition of a *pilA* construct. Lane 1 represents the WT level of PilA expression, whereas deletion of the *pilA* gene is sufficient to abolish all detectible expression (lane 2). Addition of *pilA* at the chromosomal site rescues the defect as shown in lane 3. Lane 4 represents a strain deficient in *pilA* but merodiploid for the *masABK* locus, and does not rescue the *pilA* defect. Δ*masABK* mutants lack *pilA* expression as seen in lane 5. Addition of *masABK* at the *att* site does not rescue the phenotype, as shown in lane 7, but addition of a WT copy of the *pilA* gene and necessary promoter region rescues expression of PilA in *M. xanthus* (lanes 6 and 8). Immunoblot was performed as described in materials and methods, with a polyclonal anti-pilin antibody diluted to 1∶1000.

The *pilA* construct (pSF34) restored expression of PilA protein *in vivo*. As shown in Fig. 8, WT *M. xanthus* produces a detectable amount of PilA in plate-grown culture (lane 1) and deletion of *pilA* completely abolishes expression of the type IV pilin protein (lane 2). When *pilA* was added back at the chromosomal site, expression was restored to WT levels as shown in lane 3. Addition of *mas* was not able to compensate for the Δ*pilA* defect (lane 4). In a Δ*mas* strain, pilin expression was absent (lane 5), which is compensated by addition of wild-type *pilA* as shown in lane 6. When the *mas* operon was added back to a Δ*mas* strain, production of PilA was not restored (lane 7). However when both constructs are present as is the case in lane 8, PilA is expressed at a detectable level. This confirms that addition of both *masABK* and *pilA* is responsible for the recovery of social motility and swarming behavior in *M. xanthus*.

### *masABK* is required for fruiting body formation

**Figure 9:**
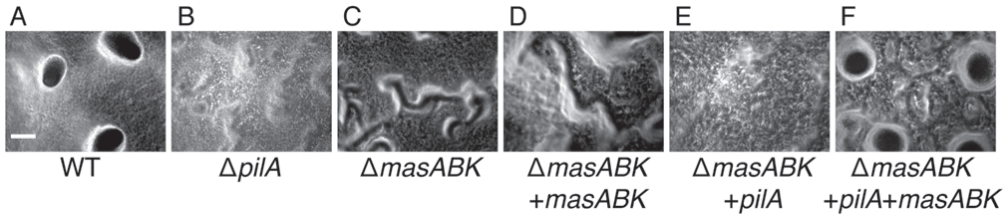
*masABK* and *pilA* are both required for fruiting body formation. *M. xanthus* strains were concentrated to a density of 5×10^9^ cells/ml and 20 µl aliquots were plated on starvation (TPM) agar as described in Methods. After 96 hours incubation, spots were photographed under using the 10× objective as seen above. The black bar in panel A denotes 100 µm. A: DK1622 (WT), B: DK10407, C: MxH2604, D: MxH2622, E: MxH2609, F: MxH2624.

Although addition of WT *pilA* was sufficient to recover 38% of WT swarming on 0.3% agar, both *masABK* and the *pilA* gene were required for the formation of fruiting bodies. After 5 d of nutrient starvation, WT *M. xanthus* forms discrete refractile fruiting bodies, as displayed in Fig. 9A. A strain with a *pilA* deletion was unable to form fruiting bodies, Fig. 9B, which indicates that PilA is critical for fruiting body formation. In a Δ*mas* strain, aggregates of cells into rudimentary mounds are visible in panel C, however no mature fruiting bodies are visible. Addition of *masABK* alone to the *mas* deletion parent failed to restore fruiting body development, Fig. 9D. Conversely, addition of *pilA* did not restore fruiting body development on starvation agar after 96 hours, as shown in Fig. 9E. Addition of both the *mas* operon and *pilA* was necessary and sufficient for normal development as shown in Fig. 9F.

As development and sporulation are related but not inseparable, germination of heat resistant spores were examined to identify if sporulation could occur in the absence of *mas*. Sporulation was similarly affected in a Δ*mas* strain as displayed in Table 3. Although no mature fruiting bodies formed in Δ*mas*, some spores were produced albeit at a reduced number (2% of the WT). Adding back *pilA* or *mas* alone failed to restore sporulation, however the strain MxH2624 displayed near WT levels of sporulation (86% of WT). Without functional copies of both *pilA* and *masABK*, no fruiting bodies formed. This result confirms that *masABK* is critical for development and sporulation of *M. xanthus* in response to starvation.

**Table 3:** Sporulation is decreased in a Δ*mas* mutant.

Strain	Heat-resistant spores	Relative to WT
WT	1×10^5^	100%
Δ*mas*	2×10^3^	2.0%
Δ*mas*+ *pilA*	3×10^3^	3.0%
Δ*mas*+ *mas*	2.4×10^3^	2.4%
Δ*mas*+ *pilA*+ *mas*	8.6×10^4^	86%

Spores were harvested from a TPM plate after 5 days of growth at 32°C and resuspended in 100 µl of TPM buffer. After 2 h incubation at 50°C, spore aliquots were diluted and plated on CTPM agar. After 3 days incubation at 32°C, CFU's from heat resistant spores were counted. All values are given as a percentage of the WT level, normalized to 100%.

### Members of the MasABK complex interact with PilA

The configuration of the Mas proteins in and associated with the membrane and the relationship between Δ*mas* and *pilAGHI* led us to speculate that Mas proteins might interact with one another and with Pil proteins to elicit the observed phenotype. To test whether these proteins could physically interact with one another, a series of pair-wise yeast two-hybrid assays were performed. Fusions were constructed between each gene of the *mas* operon and the DNA-binding domains and activation domains of the GAL4 protein using the yeast two-hybrid (Y2H) assay [44]. Both the cytoplasic domain (residues 196 to 505) of *masA* and the periplasmic domain (residues 77 to 175) of *masA* as well as *masB* were cloned into both pGAD-C1 and pGBD-C1 vectors, while *masK* was cloned into pGAD424 as described previously [15]. After transformation into haploid PJ69-4A and 4α strains, matings were performed to generate diploid strains that would contain both a pGAD and a pGBD plasmid. Presumptive positive reactions from growth on both the low and medium stringency media (SC without leucine, tryptophan and histidine, and SC without leucine, tryptophan and adenine, respectively) were tested for β-galactosidase activity.

Positive interactions drive expression of genes that allow for restoration of histidine or adenine auxotrophy or expression of β-galactosidase in the yeast host. The positive control eukaryotic transcriptional activators c-Fos and c-Jun supported growth of the yeast to 10^−6^ dilution on SC-LTH and SC-LTA media as well as the rich YPD medium dilution control. As shown in Table 4, members of the *mas* operon were capable of activating transcription of *HIS3* and *ADE2* required for histidine and adenine auxotrophy, confirming protein-protein interactions. All strains marked as +++ in Table 4 showed growth to the same dilution as the Fos/Jun control and a strong β-galactosidase activity when assayed. A (++) reaction indicates that growth was observed at a 10-fold dilution lower than the control, (+) is 100-fold lower than the positive control and growth identical to the empty vector control (pGAD-C1 and pGBD-C1 alone) is indicated as (−−). Additionally, each construct was tested for interaction with corresponding empty vector control to minimize the number of false positives. For example, pGAD-PilA did not show possible interaction with the empty pGBD vector, and pGAD-C1 alone could not interact with pGBD-PilA, as shown in Table 4. The periplasmic domain of MasA gave a positive reaction in a pair-wise reaction with both full-length MasB and MasK. MasB also activated transcription when paired with the full-length MasK protein-fusion. Although MasK gave a positive reaction with the periplasmic domain of MasA, it did not do so with the cytosolic domain of MasA.

**Table 4:** Yeast two-hybrid results reveal MasABK interactions with PilA.

pGAD-C1 insert	pGBD-C1 insert	Low (LTH)	Medium (LTA)	High (β-gal)
Fos	Jun	+++	+++	+++
No insert	No insert	−−	−−	−−
No insert	PilA	−−	−−	−−
PilA	No insert	−−	−−	−−
MasA_Cyto_	MasA_Peri_	−−	−−	−−
MasA_Cyto_	MasB	−−	−−	−−
MasA_Cyto_	PilA	+++	+++	+++
MasA_Cyto_	PilR	−−	−−	−−
MasA_Cyto_	PilS	−−	−−	−−
MasA_Peri_	MasA_Cyto_	−−	−−	−−
MasA_Peri_	MasA_Peri_	−−	−−	−−
MasA_Peri_	MasB	++	++	+++
MasA_Peri_	PilA	+++	+++	+++
MasA_Peri_	PilR	−−	−−	−−
MasA_Peri_	PilS	−−	−−	−−
MasB	MasA_Cyto_	−−	−−	−−
MasB	MasA_Peri_	++	++	+++
MasB	MasB	+	+	+
MasB	PilA	+++	+++	+++
MasB	PilR	−−	−−	−−
MasB	PilS	−−	−−	−−
MasK	MasA_Cyto_	−−	−−	−−
MasK	MasA_Peri_	++	++	+++
MasK	MasB	++	++	+++
PilA	MasA_Cyto_	++	++	+++
PilA	MasA_Peri_	+++	+++	+++
PilA	MasB	++	++	+++
PilA	PilS	++	++	+++
PilR	MasA_Cyto_	−−	−−	−−
PilR	MasA_Peri_	−−	−−	−−
PilR	MasB	−−	−−	−−
PilR	PilA	−−	−−	−−
PilR	PilS	+++	+++	+++
PilS	MasA_Cyto_	−−	−−	−−
PilS	MasA_Peri_	−−	−−	−−
PilS	MasB	−−	−−	−−
PilS	PilA	++	++	+++
PilS	PilR	+++	+++	+++

Yeast two-hybrid experiments were performed as described in Materials and Methods. Low stringency media is synthetic complete agar without Leu, Trp, and His (LTH); medium stringency media is synthetic complete agar without Leu, Trp, and Ade (LTA).

**Figure 10:**
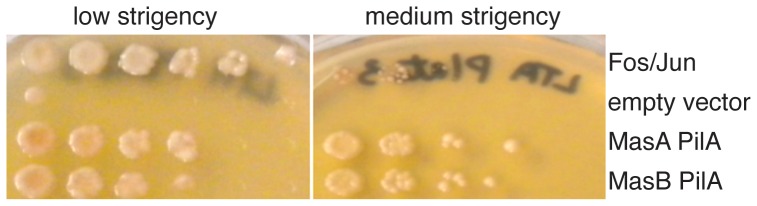
MasAB-PilA interaction drives transcription from the GAL promoter in a Y2H assay. Diploid yeast strains containing both a pGAD-C1 and pGBD-C1 derived vectors. Dilutions of overnight culture were plated on a low stringency medium (SC-LTH) Panel A, and medium stringency medium (SC-LTA), Panel B as described in Materials and Methods. Row 1: *FOS/JUN* (+) control, Row 2: Empty vector (pGAD, pGBD negative control), Row 3: pGAD*masA*, pGBD*pilA*, Row 4, pGAD*masB*, pGBD*pilA*.

As swarm and development data indicated a relationship between the function of PilA and MasABK, potential interactions between Mas proteins and PilA were examined using the Y2H assay. Yeast cells carrying pSF35 *(masA* – periplasmic domain) and pSF42 (*pilA*) grew well on medium lacking either His or Ade. In the presence of X-gal, blue pigmentation was observed, showing that MasA and PilA interacted as strongly as the Fos/Jun control. Additional pairings between the cytoplasmic portion of MasA and PilA showed similar results. A similar interaction could be observed between MasB and PilA, but no interactions were identified between PilA and MasK as shown in Fig. 10, a representative dilution series on SC-Leu, -Trp, -His. The top row shows the Fos/Jun positive control showing robust growth, while the second row demonstrates the inability of empty vectors to activate transcription of the *HIS3* gene conferring histidine auxotrophy. Growth was observed in strain YSF13, however, using the MasA-periplasmic pGAD-C1 plasmid pSF35 and pSF42, the PilA- pGBD-C1 fusion, as shown in row 3. Additionally, growth was robust in strain YSF31 (row 4 of MasB section), pSF30 (MasB in pGAD-C1) and pSF42 (PilA in pGBD-C1), indicating a strong interaction between these protein pairs.

As MasA and B were shown to interact with PilA, the potential for interactions with the PilS and PilR TCS was investigated. PilS is the sensor histidine kinase demonstrated to affect PilA expression in *M. xanthus* while PilR is the response regulator in this TCS [9]. Interaction between PilS and PilR was confirmed at all three levels of the Y2H assay as shown in Table 4. Additionally, interaction between PilA and PilS was observed to occur, though activation was observed at a lower dilution (10-fold decrease) relative to the Fos/Jun control. PilR did not directly interact with PilA, however; this was expected as PilR binds to the region of the DNA upstream of PilA rather than interacting with the protein directly. No interaction was observed between either member of the PilS/PilR TCS or any of the Mas protein fusions assayed. Growth was not observed on either of the restrictive media, and no β-galactosidase activity was detected in the assay for any Mas-PilS/R pairing. MasAB must interact with PilA in a manner functionally, temporally or spatially removed from either PilR or PilS.

## Discussion

The *masABK* operon is transcribed divergent to the *mglBA* operon, which encodes a GAP-like protein and GTPase, respectively. Loss of MglA, the GTPase, renders cells nonmotile and models propose that MglA coordinates cell reversal with the activities of the *M. xanthus* A- and S-motility systems. It is unclear what signal(s) stimulates cells to reverse and exactly how the message to reverse is communicated to MglA and the two gliding systems.

Previous work demonstrated a role for MasK in restoring social motility to a nonmotile *mglA* mutant and established a genetic link between the two genes [15]. Based on the organization of the *mas* operon, we predicted that *masA* and *masB*, the two genes that precede *masK* might also play a role in gliding. *in silico* data suggested that Mas proteins reside in the membrane and our experimental results confirmed that MasA is an integral membrane protein and MasK is associated with the membrane at the leading pole. MasB possesses the necessary signal sequence for an inner-membrane bound lipoprotein, but no predicted transmembrane domains or conserved domain motifs were noted. While a number of putative IM lipoproteins were identified in *M. xanthus* by LC/MS-MS [45] with a consensus of LTGCK, MasB (_15_VAGCD_19_) lacks the obvious consensus +2 lysine found in many inner membrane lipoproteins. In *E. coli*, however, it has been shown that one aspartate immediately following the lipid-anchored cysteine is sufficient for targeting to the inner membrane during translocation to the periplasm [46]. The presence of not one, but three aspartate residues after the conserved cysteine (_18_CDDD_21_) strongly favors the model that MasB is anchored to the inner membrane rather than the outer membrane. All three genes were deleted to establish a baseline phenotype and avoid intermediate phenotypes due to alterations in balance of products of critical gliding components in *M. xanthus* [47].

Deletion of *masABK* created MxH2604, a yellow pigmented strain with significantly diminished swarming, S-gliding motility, and development of fruiting bodies and spores. Although swarming on both hard and soft agar surfaces initially gave the impression that both A-motility and S-motility were affected by loss of *mas*, microscopic analysis showed that the A-motility motor was unimpeded in the mutant. In contrast, the S-motility motor was nonfunctional. The motor for S-motility in *M. xanthus* is type IV pili, the structural subunit for which is encoded by *pilA* and a plethora of genes including those encoding assembly or disassembly machinery (*pilG, pilH, pilI, pilT, pilB, pilC, pilQ* or *tgl*), pilin biosynthesis clusters, regulators (*pilS, pilR*), exopolysaccharide biosynthesis (*eps*), and fibril formation (*dif* and *fib* genes) [39], [48], [49].

Given that over 100 genes are known to be involved in S-motility, it was surprising that expression of only one *bona fide* S-motility operon – *pilAGHI* – was decreased in the Δ*mas* mutant. Consistent with this finding, PilA protein was not detected in the mutant. The absence of PilA undoubtedly contributed to the observed phenotype in Δ*mas*, which mirrors that of a *pilA* mutant except for the fact that EPS is decreased significantly in the *pilA* mutant (EPS-), but less so in the Δ*mas* mutant. In this regard, the Δ*mas* mutant, which is, in essence, a Δ*mas pilA* double mutant, is phenotypically similar to the EPS+ *pilA pilB* double mutant [50]. Another surprise was that expression of a second operon (MXAN_0360–0364) that encodes proteins homologous to pilin biogenesis proteins also was decreased significantly in the Δ*mas* mutant. Little is known about these genes, but they do not appear to be essential for S-motility. While being homologous to TFP biogenesis proteins, these proteins are also similar to pseudopilin-like proteins which raises the possibility that they play ancillary or redundant roles in secretion or processing of products needed for motility or swarming or exhibit phenotypes under conditions that are not observed in a controlled laboratory environment.

MasABK proteins do not contain any DNA binding motifs, so we looked to other sources to understand why the *pilAGHI* operon was depressed in Δ*mas*. The two component system PilS/PilR has been shown to regulate expression of PilA in *M. xanthus* [9]. PilR positively regulates PilA expression, while PilS negatively affects PilA expression. Additionally, PilA has been shown to autoregulate expression. As the expression of the *pilSR* operon remained unchanged in Δ*mas*, another explanation was needed to explain the dramatic decrease in *pilA* mRNA. We considered that the function of MasABK might be to recognize and present PilA for interaction with PilS, which is predicted to be an integral membrane protein. In the absence of MasABK, PilA may be unable to properly interact with PilS. Without this signal, PilS would repress transcription through its interacting partner PilR, and lead to repression of *pilA* and the loss of both social motility and development. However, addition of *masABK* to the Δ*mas* mutant should have restored the complex. Instead the plasmid-borne *masABK*, integrated at *attB*, did not rescue the motility or development defects of the *Δmas* mutant (Fig. 6 and 9). The *masABK* transcript was produced but it appeared that complementation failed because expression of *pilA* was not restored. Although the reasons for this are not clear, it implicates other components, such as the LuxR-family regulator, the cryptic pilin gene cluster and even myxalamide, in the Δ*mas* phenotype and regulation of *pilAGHI*. The increase in myxalamide gene expression might contribute to the diminished swarming seen on both hard and soft agar surfaces. *M. xanthus* produces a class of lipopeptides that stimulate swarming (R. Muller, personal communication). Structurally, myxalamide is similar to lipopeptides but instead of stimulating swarming it may inhibit swarming when its expression is increased. This might account for the Δ*mas* swarming defect on soft and hard surfaces.

A partial answer to the puzzle was obtained when addition of a second copy of *pilA* to the Δ*mas* mutant (without added *masABK*) improved S-dependent swarming at high-cell density and of gliding in methylcellulose. In other words, two functional copies of *pilA* helped overcome the loss of MasABK. However, *pilA* expression in the absence of *masABK* was not sufficient to induce fruiting body development. Reintroduction of *masABK* along with a second copy of *pilA* restored motility, fruiting and sporulation leading to the conclusion that *masABK* is crucial for fully functional motility and starvation responses in *M. xanthus*.

The Mas proteins reside in the membrane (MasA, K) or are anchored to the membrane (MasB) and their strong interactions support the model that they form a polar membrane complex that involves PilA (Fig. S4). Based on the Y2H results, it was possible to speculate that sensing of pre-PilA or processed PilA by MasABK transmits a signal to cytosolic regulators, such as MglA, the LuxR-family protein identified in this work or the response regulator DigR [51], or sequesters PilA to attenuate negative regulation through the PilS/R system. Moreover, the finding that the loss of MasABK suppresses the EPS defect associated with a *pilA* mutant argues that the Mas protein complex plays a role in maintaining the balance between pilin and EPS.

Although *pilA* did not appear to be the target of a heritable mutation as determined by sequencing, we cannot rule out the possibility that epigenetic modification, such as DNA methylation, may affect native *pilAGHI* expression in the Δ*masABK* mutant. Alternatively, a second mutation in PilS/PilR, the two-component system known to regulate *pilAGHI* or in another location in the chromosome may diminish *pilA* expression. The paradox of *pilA* expression in Δ*mas* requires further investigation and will likely yield vital information about regulation of gene expression in *Myxococcus xanthus*.

## Materials and Methods

### Cultivation of strains

*M. xanthus* strains were cultivated at 32°C in CTPM broth or CTPM with 1.5% agar plates. *E. coli* strains were grown on LB medium at 37°C. When appropriate, kanamycin was added to a final concentration of 40 µg/ml, ampicillin to 100 µg/ml, and zeocin to 25 µg/ml for *E. coli* and 50 µg/ml for selection of resistant *M. xanthus* strains. Unless otherwise specified, *M. xanthus* strains were grown to a concentration of 5×10^8^ before assay or plating. Plasmid constructs, bacterial strains and PCR reactions are listed in Table S1.

### Determination of membrane topology using alkaline phosphatase

MasA was cloned into pCR Blunt II-TOPO (Invitrogen) with a p*lac* promoter and *E. coli* ribosomal binding site (RBS) fused upstream, called pSF8. The resulting plasmid was cut with PstI or MluI, and the *phoA* gene from pUT-mini*Tn5*-PhoA was cloned in at one of the restriction sites. After confirmation of the insertion by restriction digest, these plasmids were electroporated into DH5α (*phoA* negative strain) to eliminate *phoA* background activity. Both DH5α and DH5α with pSF8 were used as negative controls. Strains containing plasmids with PhoA were diluted in dH_2_O in a 96 well plate, and stamped on LB with X-P (5-Bromo-4-chloro-3-indolyl phosphate, Sigma-Aldrich). After 16 hours incubation at 37C, the plate was removed and photographed in order to minimize background BCIP degradation.

### Generation of *mas* deletion mutant

Experiments in other labs revealed that either a transposon insertion or an in-frame deletion of the *masK* gene yielded viable mutants with no discernible motility phenotype (personal communication S. Inouye, L. Sogaard-Andersen), therefore a full operon deletion was constructed. The *mglBA* operon was amplified and cloned into the *Bam*HI site of pBJ114, while the downstream 1.5 kb *recR* fragment was cloned into a *Hin*dIII-*Pst*I site. After confirmation of this construct, the resulting plasmid pGF94 was electroporated into DK1622 (WT) and transformants were selected for by kanamycin resistance. Merodiploid transformants were grown in liquid culture for 30 days with daily dilutions before plating on 2% galactose to select against the pGF94 insertion using the *galK* counterselectable marker. Kanamycin sensitive colonies with altered motility phenotype were subjected to colony PCR analysis to check for the pGF94 insert construct and absence of *mas* gene products. Regions including the *mglBA* and *recR* operons were cloned adjacent to one another in pBJ114, a plasmid that carried kanamycin resistance and sensitivity to galactose to form pGF94. After electroporation of pGF94 into a wild-type DK1622 strain, kanamycin-resistant merodiploids were obtained. This merodiploid strain was grown for one month in the absence of antibiotic selection and then plated on CTPM +2% galactose. PCR was performed using primer 802 and 807 in order to identify the pGF94 construct in Δ*mas* genomic DNA, while amplification of the *masA* gene required primers 804 and 805. 100 ng of genomic DNA was used as a template in the PCR reactions. 5 µl of the PCR reactions were loaded onto a TAE 1% agarose gel and electrophoresed at 100 V for 45 minutes.

Complementation of deletions was achieved by cloning appropriate fragments into pCR Blunt II-TOPO (Invitrogen). pSF26 was constructed by cloning a fragment of genomic DNA from 300 bp of *mglB* to the 3′ end of *masK* as described in Table S1.

### Motility, development, and dye-binding assays

Swarming assays were performed as previously described [1]. Nine individual swarms were measured and averaged. The standard deviations of the calculated areas are shown as error bars. Development assays were performed as previously described [14] and fruiting bodies were photographed using a Nikon SMZ-U microscope under 100x total magnification. Single-cell adventurous motility was assayed by plating on CTPM pad with 1% agarose and then covered with a glass coverslip and incubated at 32C for 30 min. Alternatively, single cell social motility was assayed by pipetting overnight cultures into a silicone gasket adhered to a microscope slide. After 20 min incubation at room temperature, the liquid was removed by micropipettor, and cells were overlaid with a 0.5% methylcellulose, 0.5× CTPM mixture. After overlaying with a coverslip, these were incubated at 32C for 30 min.

Microscopy was performed at 200× magnification on the Nikon Microphot-FXA microscope, taking photographs every 30 seconds for 30 min. Cells were tracked using the automated software on Metamorph, and 50 cells were tracked for average velocity and reversal frequency calculations. Reversals were determined using the MotilityMacro v2.2 [52].

Dye binding and cohesion analyses were performed as previously described [53]–[55].

### Microarray analysis

A custom Roche NimbleGen 12-plex microarray was designed for *Myxococcus xanthus* based on a consensus sequence from mapping paired end 91 bp Illumina sequence data from the National Genome Research Center for four clones to the reference genome (dk_1622). The microarray contained 137,799 60-mer probes tiled across the genome, with an average spacing of 65 bp, and representing a possible 6,935 expressed transcripts (probe sets). Probe sets were defined as all probes that mapped to a single gene. Double-stranded cDNAs were synthesized using Invitrogen's SuperScript cDNA Synthesis Kit with the standard protocol using random hexanucleotide primers (Invitrogen, Carlsbad, CA). RNA was harvested from WT, MxH2604 and MxH2627 grown in liquid culture to a density of 5×10^8^ cells/ml as described elsewhere [47]. cDNAs were fluorescently labeled with Cy3 using the standard NimbleGen Gene Expression Analysis protocol. All samples were hybridized to a single 12-plex chip to reduce technical noise. An 18-hour hybridization was conducted at 42 degrees in a NimbleGen Hybridization System 4 chamber (NimbleGen, Madison, WI). The chip was washed and then scanned on a Roche MS200 scanner. Finally, probe signal intensities were extracted using Roche Nimblegen's NimbleScan Software v2.6. Samples with adjusted p values of <0.05 from the independent array runs (triplicate samples of three biological replicates) were considered statistically significant. All analyses were done in the R programming language [48], raw intensity data were read in and checked for quality using the Limma package [49]. The data were then background corrected using background subtraction and the arrays were normalized with quantile normalization [50]. A simple linear model was fit to the data and variances were corrected using an empirical Bayes approach [51] from the bioconductor limma package. Results were corrected for multiple testing using the false discovery rate procedure of Benjamini and Hochberg (BH) and considered significant if their BH adjusted p-values were <0.05 [52].

### qRT-PCR analysis

Total RNA was harvested from *M. xanthus* strains using Trizol RNA isolation procedure. After RNA was redissolved in DEPC-H_2_O, samples were treated with DNaseI (Roche) for 1 hr at 37C. Phenol-chloroform extraction was performed to isolate RNA. 500 ng of mRNA was used to synthesize cDNA using the Superscriptase II first-strand kit (Invitrogen) with a random hexamer oligonucleotide primer (Roche).

qRT-PCR was performed on an ABI 7900HT Real-Time PCR using a SYBR Green detector. Genes of interest were normalized against the 16 s rRNA probe, diluted 1∶2500, while target genes were probed at a 1∶25 dilution of the original cDNA. Relative quantitation was performed in triplicate for each sample, and results represent the expression of selected genes compared to the WT strain. Primers used are listed in Table S1.

Semi-quantitative PCR was additionally performed on complementing strains in order to confirm expression of *masB* from strains with pSF26 (*masABK att-int*). PCR was performed using primers specific to *masB* and the *16S* rRNA to compare transcript production. Undiluted cDNA equivalent to 50 ng of total RNA was used as the primary dilution for *masB* screening, though dilutions of 1∶10 and 1∶100 were also used. 16 s rRNA was probed at dilutions of 1∶2500, 1∶25,000 and 1∶250,000 from the original cDNA template. PCR was performed using the Platinum Pfx kit (Invitrogen) for a total reaction size of 50 µl. 5 µl of the semi-quantitative PCR reactions were run on a 1% agarose TAE gel and electrophoresed at 100 V for 35 min. A 1KB+ ladder (NEB) was used to determine correct template size and the resulting gel was visualized using a gel-doc system.

### Immunoblot analysis

Aliquots of 5×10^9^ cells/ml were frozen in TPM with Roche Complete Mini EDTA-free protease inhibitor cocktail. Upon thawing, SDS loading dye buffer was added to 1X final concentration. After boiling at 95C, whole cell extracts were cooled to room temperature and 15 µl of each sample was applied to a 12.5% SDS PAGE gel. Samples were electrophoresed for ∼1.5 hr at 40 mA in a mini-Protean-II (Bio-Rad) apparatus, then transferred to a PVDF membrane for 45 min at 200 mA. A polyclonal antibody raised against PilA was diluted 1∶1000 in 3% BSA in a Tris-HCl, NaCl, pH 7.5 blocking solution with 0.1% Tween-20. Polyclonal anti-MglA antibodies were diluted at 1∶1000 in blocking solution. Detection of primary antibodies was performed using an anti-rabbit IR800 nm tagged goat secondary antibody, (Rockland). Scans were performed on the LiCor Odyssey.

### Yeast Two-Hybrid Assays

Plasmids containing fusions with the Gal4 activation domain (pGAD-C1) and DNA binding domain (pGBD-C1) were transformed into the strains PJ69-4A and PJ69-4α respectively using the LiAc method [56] and selected for on synthetic complete lacking leucine and tryptophan, respectively [15]. Haploid strains were grown to OD_600_ of 1 and 10 µl each of cultured A and α strains were pipetted onto a YPD plate. After overnight incubation at 30C, the mating reaction was streaked onto SC-LT (synthetic complete agar without leucine or tryptophan) to select for diploid strains.

Diploid strains were cultured overnight at 30C with shaking in YPD liquid, then normalized to an OD_600_ of 1. Cells were washed twice with dH_2_O, then serially diluted to 10^−6^ in a 96 well plate. 3 µl of each dilution was pipetted onto each of three agars: YPD (permissive), SC-LTH (low), and SC-LTA (medium stringency). Plates were incubated at 30C for 2–5 days to allow for full growth. To test for β-galactosidase activity, the flash-freezing lift-off assay was performed as previously described [15].

## Supporting Information

Figure S1 The Δ*mas* strain contains the pGF94 construct but lacks *masA*. The top panel shows amplification of the pGF94 construct in the original plasmid, as well as the *mas* mutant MxH2604, but not in the WT. The lower panel shows amplification of a probe specific to the *masA* gene, and is detected in the WT, but not in either the plasmid pGF94 or the *mas* deletion strain. Amplification was performed as described in Materials and Methods.
(DOCX)

Figure S2 Δ*mas* still produces WT levels of MglA *in vivo*. In order to determine if expression from the *mgl* promoter was affected by deletion of the *mas* operon, whole cell extract was probed with anti-MglA antibody as described in Materials and Methods. While a negative control *mgl* mutant strain failed to produce detectible amounts of MglA, both the WT and *mas* mutant produced detectible amounts of MglA, showing that MglA production was not visibly affected by a *mas* deletion.
(DOCX)

Figure S3 The extracellular matrix is altered in Δmas. A. Congo red and trypan blue were added to aliquots of cells as described in Black and Yang (30). The percent of dye bound to cells was the difference in OD from the no cell control and residual dye remaining in supernatant after preincubation with WT or Δmas. Blue bars represent the amount of trypan blue bound by *M. xanthus* while red bars indi- cate the amount of Congo red bound. B. Cohesion assays measure the ability of cells to aggregate during incubation in cohesion buffer as described by Dana and Shimkets (49). Optical density decreases as the cells aggregate and precipitate. WT  =  solid line (–) while Δmas  =  dashed line (- - -).
(PDF)

Figure S4 Model of MasABK, MglA, and PilASR. Pre-PilA (purple) may interact with MasA and mature PilA protein in the periplasm may interact with both MasA (green) and MasB (blue). When pre-PilA remains at high levels in the inner membrane or cytoplasm prior to processing, interaction with PilS (turquoise) results in inhibition of PilR (fuchsia), leading to a decrease in transcription of the *pilAGHI* operon. EPS is needed to stimulate retraction of pili; loss of PilA blocks production of EPS. The MasB-MasK (red) interaction might alter the MasK-MglA (orange) interaction, leading to release of MglA from the leading pole.
(EPS)

Table S1 Strains and molecular reagents.
(DOCX)
