# Supplementary material for: MasABK Proteins Interact with Proteins of the Type IV Pilin System to Affect Social Motility of Myxococcus xanthus
Source: PLoS One. 2013 Jan 16;8(1):e54557. doi: 10.1371/journal.pone.0054557 (PMC3546991; doi:10.1371/journal.pone.0054557)
Supplement: Figure S1 — The Δmas strain contains the pGF94 construct but lacks masA. The top panel shows amplification of the pGF94 construct in the original plasmid, as well as the mas mutant MxH2604, but not in the WT. The lower panel shows amplification of a probe specific to the masA gene, and is detected in the WT, but not in either the plasmid pGF94 or the mas deletion strain. Amplification was performed as described in Materials and Methods. (DOCX) [file pone.0054557.s001.docx]

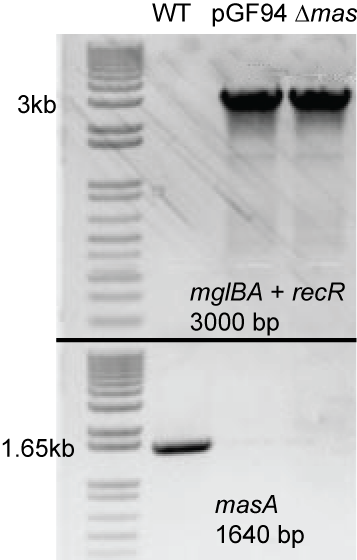


Supplemental Fig. S1: The Δ*mas* strain contains the pGF94 construct but lacks *masA.* The top panel shows amplification of the pGF94 construct in the original plasmid, as well as the *mas* mutant MxH2604, but not in the WT. The lower panel shows amplification of a probe specific to the *masA* gene, and is detected in the WT, but not in either the plasmid pGF94 or the *mas* deletion strain. Amplification was performed as described in Materials and Methods.
