# Supplementary material for: MasABK Proteins Interact with Proteins of the Type IV Pilin System to Affect Social Motility of Myxococcus xanthus
Source: PLoS One. 2013 Jan 16;8(1):e54557. doi: 10.1371/journal.pone.0054557 (PMC3546991; doi:10.1371/journal.pone.0054557)
Supplement: Figure S2 — Δmas still produces WT levels of MglA in vivo. In order to determine if expression from the mgl promoter was affected by deletion of the mas operon, whole cell extract was probed with anti-MglA antibody as described in Materials and Methods. While a negative control mgl mutant strain failed to produce detectible amounts of MglA, both the WT and mas mutant produced detectible amounts of MglA, showing that MglA production was not visibly affected by a mas deletion. (DOCX) [file pone.0054557.s002.docx]

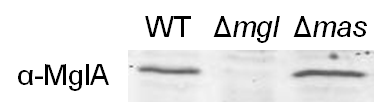


Supplemental Fig. S2:Δ*mas* still produces WT levels of MglA *in vivo.* In order to determine if expression from the *mgl* promoter was affected by deletion of the *mas* operon, whole cell extract was probed with anti-MglA antibody as described in Materials and Methods. While a negative control *mgl* mutant strain failed to produce detectible amounts of MglA, both the WT and *mas* mutant produced detectible amounts of MglA, showing that MglA production was not visibly affected by a *mas* deletion.
