# Supplementary material for: MasABK Proteins Interact with Proteins of the Type IV Pilin System to Affect Social Motility of Myxococcus xanthus
Source: PLoS One. 2013 Jan 16;8(1):e54557. doi: 10.1371/journal.pone.0054557 (PMC3546991; doi:10.1371/journal.pone.0054557)
Supplement: Figure S3 — The extracellular matrix is altered in Δmas. A. Congo red and trypan blue were added to aliquots of cells as described in Black and Yang (30). The percent of dye bound to cells was the difference in OD from the no cell control and residual dye remaining in supernatant after preincubation with WT or Δmas. Blue bars represent the amount of trypan blue bound by M. xanthus while red bars indi- cate the amount of Congo red bound. B. Cohesion assays measure the ability of cells to aggregate during incubation in cohesion buffer as described by Dana and Shimkets (49). Optical density decreases as the cells aggregate and precipitate. WT = solid line (–) while Δmas = dashed line (- - -). (PDF) [file pone.0054557.s003.pdf]

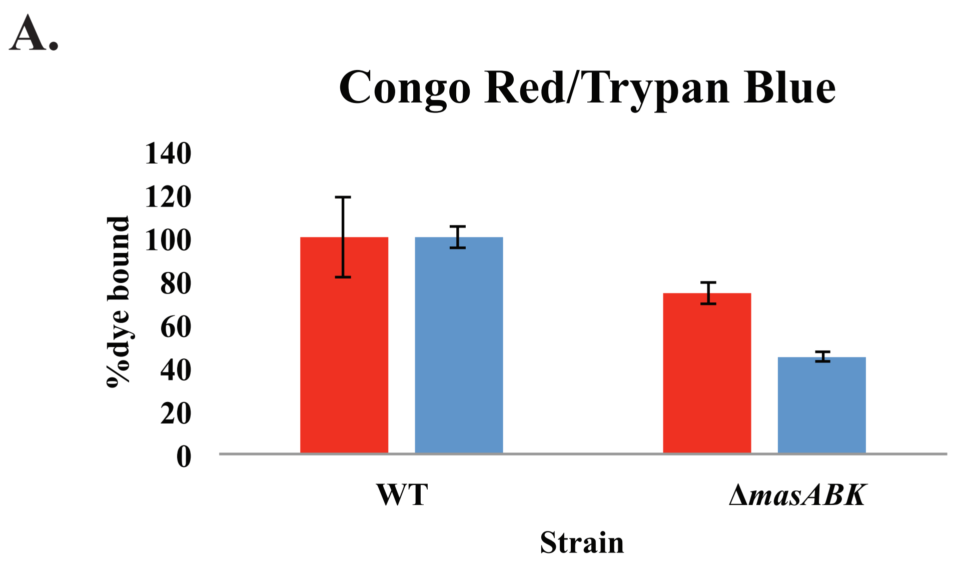

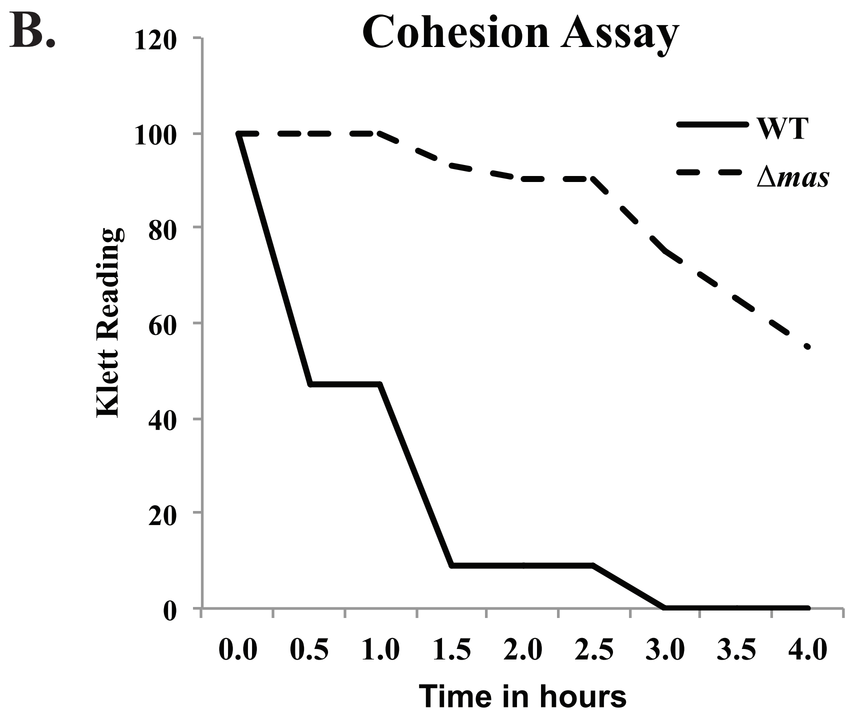

Figure S3: The extracellular matrix is altered in Δ*mas*. A. Congo red and trypan blue were added to aliquots of cells as described in Black and Yang (30). The percent of dye bound to cells was the difference in OD from the no cell control and residual dye remaining in supernatant after preincubation with WT or Δ*mas*. Blue bars represent the amount of trypan blue bound by *M. xanthus* while red bars indicate the amount of Congo red bound. B. Cohesion assays measure the ability of cells to aggregate during incubation in cohesion buffer as described by Dana and Shimkets (49). Optical density decreases as the cells aggregate and precipitate. WT = solid line (–) while Δ*mas* = dashed line (- - -)
