# Supplementary material for: MasABK Proteins Interact with Proteins of the Type IV Pilin System to Affect Social Motility of Myxococcus xanthus
Source: PLoS One. 2013 Jan 16;8(1):e54557. doi: 10.1371/journal.pone.0054557 (PMC3546991; doi:10.1371/journal.pone.0054557)
Supplement: Table S1 — Strains and molecular reagents. (DOCX) [file pone.0054557.s005.docx]

Table S1: Strains and molecular reagents

| A: Plasmids and vectors | | | | | | | | | |
| --- | --- | --- | --- | --- | --- | --- | --- | --- | --- |
| Plasmid name | | Genotype | | | Construction/Reference | | | |  |
| pACW1 | | *masA*-*phoA* fusion | | | Cloning of *phoA* from pACW2 into pSF8 | | | |  |
| pACW2 | | *phoA-Mlu*I ends | | | PCR 100 in TOPO | | | |  |
| pACW3 | | *phoA*-*Pst*I ends | | | PCR 101 in TOPO | | | |  |
| pAGS164 | | *aglZ* disruption plasmid | | | [1] | | | |  |
| pBJ114 | | *galK* Kan^R^ | | | [2] | | | |  |
| pBT17 | | GAL4-AD-MasK | | | [3] | | | |  |
| pCR Blunt II-TOPO | | Kan^R^, Zeo^R^ | | | Invitrogen | | | |  |
| pGAD-C1 | | GAL4-AD Amp^R^, Leu^+^ | | | [4] | | | |  |
| pGBD-C1 | | GAL4-DBD Amp^R^, Trp^+^ | | | [4] | | | |  |
| pGF27 | | *Bam*HI *att-int* | | | This work | | | |  |
| pGF90 | | *mglBA* operon | | | PCR 102 in TOPO | | | |  |
| pGF93 | | *recR* operon | | | Cloned into pBJ114 | | | |  |
| pGF94 | | *mglBA recR* in pBJ114 Kan^R^, *galK* | | | *mglBA* cloned into pGF93 | | | |  |
| pPLH512 | | *mcherry* | | | Clone of PCR 55 in pCR-Blunt II TOPO | | | |  |
| pSF6 | | *masK*-*mcherry* fusion | | | Clone of PCR 95 in pCR-Blunt II TOPO | | | |  |
| pSF8 | | P*lac* *masA* | | | Clone of PCR 111 in pCR-Blunt II TOPO | | | |  |
| pSF20 | | *masA*-*phoA* at PstI site | | | Cloning of *phoA* from pACW3 into pSF8 | | | |  |
| pSF25 | | *masABK* | | | Clone of PCR 103 in TOPO | | | |  |
| pSF26 | | *masABK* + *att-int* | | | SpeI-DraI fragment of pGF27 + *Xba*I-*Dra*I fragment of pSF25 | | | |  |
| pSF29 | | GAL4-DBD-MasB | | | In-fusion cloning of PCR 104 in pGBD-C1 | | | |  |
| pSF30 | | GAL4-AD-MasB | | | In-fusion cloning of PCR 104 in pGAD-C1 | | | |  |
| pSF33 | | *pilA* Kan^R^, Zeo^R^ | | | Clone of PCR 105 in TOPO | | | |  |
| pSF34 | | *pilA* Kan^R^, Zeo^S^ | | | *Dra*I-*Hinc*II – self-ligated | | | |  |
| pSF35 | | GAL4-AD-MasA_77-175_ | | | In-fusion cloning of PCR 106 in pGAD-C1 | | | |  |
| pSF36 | | GAL4-DBD-MasA_77-175_ | | | In-fusion cloning of PCR 106 in pGBD-C1 | | | |  |
| pSF37 | | GAL4-AD-MasA_201-504_ | | | In-fusion cloning of PCR 107 in pGAD-C1 | | | |  |
| pSF38 | | GAL4-DBD-MasA_201-504_ | | | In-fusion cloning of PCR 107 in pGBD-C1 | | | |  |
| pSF39 | | GAL4-AD-PilA_19-221_ | | | In-fusion cloning of PCR 108 in pGAD-C1 | | | |  |
| pSF40 | | GAL4-AD-PilR | | | In-fusion cloning of PCR 109 in pGAD-C1 | | | |  |
| pSF41 | | GAL4-AD-PilS | | | In-fusion cloning of PCR 110 in pGAD-C1 | | | |  |
| pSF42 | | GAL4-DBD-PilA_19-221_ | | | In-fusion cloning of PCR 108 in pGBD-C1 | | | |  |
| pSF43 | | GAL4-DBD-PilR | | | In-fusion cloning of PCR 109 in pGBD-C1 | | | |  |
| pSF44 | | GAL4-DBD-PilS | | | In-fusion cloning of PCR 110 in pGBD-C1 | | | |  |
| pSF49 | | p*pilA + pilA* from WT | | | Clone of PCR 112 in pCR-Blunt II TOPO | | | |  |
| pSF50 | | p*pilA + pilA* from Δ*mas* | | | Clone of PCR 113 in pCR-Blunt II TOPO | | | |  |
| Tn*phoA* | | Tn*phoA* Amp^R^, Kan^R^ | | | [[47](#_ENREF_47)] | | | |  |
| *E. coli* strains | | Genotype | | | | | | Reference,  Source |  |
| Top 10 | | F^-^ *mcr*A Δ(*mrr-hsd*RMS-*mcr*BC) Φ80*lac*ZΔM15 Δ*lac*X74 *rec*A1 *ara*D139 Δ(*ara-leu*)7697 *gal*U *gal*K *rps*L (Str^R^) *end*A1 *nup*G | | | | | | Invitrogen |  |
| DH5α | | F^-^ φ80*lac*ZΔM15 Δ(*lac*ZYA-*arg*F)U169 *rec*A1 *end*A1 *hsd*R17(r_k_^-^, m_k_^+^) *pho*A *sup*E44 *thi*-1 *gyr*A96 *rel*A1 *ton*A | | | | | | Invitrogen |  |
| Stellar cells | | F^-^ *endA1, supE44, thi-1, recA1, relA1, gyrA96, phoA, Φ80d lacZ*Δ *M15,* Δ*(lacZYA-argF)U169,* Δ*(mrr-hsdRMS-mcrBC), ΔmcrA, λ-* | | | | | | Clontech |  |
| *M. xanthus* strains | | | | | | | |  |  |
| DK1622 | | WT Kan^S^ | | | | | | [5] |  |
| DK6204 | | Δ*mglBA* Kan^S^ | | | | | | [[5](#_ENREF_48)] |  |
| DK10407 | | Δ*pilA* Kan^S^ | | | | | | [6] |  |
| MxH2604 | | Δ*masABK* Kan^S^ | | | | | | This Work |  |
| MxH2545 | | WT + pGF94 *masABK* deletion precursor Kan^R^ *gal*K | | | | | | This Work |  |
| MxH2608 | | WT + *pilA* (pSF34) Kan^R^ | | | | | | This Work |  |
| MxH2620 | | WT + *masABK* (pSF26) *att-int* Kan^R^, Zeo^R^ | | | | | | This Work |  |
| MxH2623 | | WT + *pilA* + *masABK* Kan^R^, Zeo^R^ | | | | | | This Work |  |
| MxH2610 | | DK10407 + *pilA* Kan^R^ | | | | | | This Work |  |
| MxH2621 | | DK10407 + *masABK* *att-int* Kan^R^, Zeo^R^ | | | | | | This Work |  |
| MxH2609 | | MxH2604 + *pilA* Kan^R^ | | | | | | This Work |  |
| MxH2622 | | MxH2604 + *masABK* *att-int* Kan^R^, Zeo^R^ | | | | | | This Work |  |
| MxH2624 | | MxH2604 + *pilA* + *masABK* *att-int* Kan^R^, Zeo^R^ | | | | | | This Work |  |
| MxH2627 | | MxH2604 + pSF25 | | | | | | This Work |  |
| MxH2639 | | WT + pSF6 *masK-mcherry* Kan^R^, Zeo^R^ | | | | | | This Work |  |
| *S. cerevisiae* strains | | | | | | | |  |  |
| PJ69-4A | | MATa *trp*1-901, *leu*2-3, 112 *ura*3-52 *his3*-200 Δ*gal4* Δ*gal80* LYS2::GAL1-HIS3 GAL2-ADE2 *met*2::GAL7-*lacZ* | | | | | | [4] |  |
| PJ69-4α | | MATα *trp*1-901, *leu*2-3, 112 *ura*3-52 *his*3-200 Δ*gal4* Δ*gal80* LYS2::GAL1-HIS3 GAL2-ADE2 *met*2::GAL7-*lacZ* | | | | | | [4] |  |
| Fos/Jun | | MATa/α *ibid.* Fos/Jun | | | | | | [3] |  |
| Empty Vector | | MATa/α *ibid.* pGAD-C1, pGBD-C1 | | | | | | This work |  |
| YSF1 | | MATa/α *ibid.* pBT17, pSF38 | | | | | | This work |  |
| YSF2 | | MATa/α *ibid.* pSF35, pSF29 | | | | | | This work |  |
| YSF3 | | MATa/α *ibid.* pSF37, pSF29 | | | | | | This work |  |
| YSF4 | | MATa/α *ibid.* pSF37, pSF38 | | | | | | This work |  |
| YSF5 | | MATa/α *ibid.* pSF35, pSF36 | | | | | | This work |  |
| YSF6 | | MATa/α *ibid.* pSF35, pSF38 | | | | | | This work |  |
| YSF7 | | MATa/α *ibid.* pSF37, pSF36 | | | | | | This work |  |
| YSF8 | | MATa/α *ibid.* pSF30, pSF38 | | | | | | This work |  |
| YSF9 | | MATa/α *ibid.* pSF30, pSF36 | | | | | | This work |  |
| YSF10 | | MATa/α *ibid.* pBT17, pSF36 | | | | | | This work |  |
| YSF11 | | MATa/α *ibid.* pSF30, pSF29 | | | | | | This work |  |
| YSF12 | | MATa/α *ibid.* pBT17, pSF29 | | | | | | This work |  |
| YSF13 | | MATa/α *ibid.* pSF35, pSF42 | | | | | | This work |  |
| YSF14 | | MATa/α *ibid.* pSF37, pSF42 | | | | | | This work |  |
| YSF15 | | MATa/α *ibid.* pSF35, pSF44 | | | | | | This work |  |
| YSF16 | | MATa/α *ibid.* pSF37, pSF44 | | | | | | This work |  |
| YSF17 | | MATa/α *ibid.* pSF35, pSF43 | | | | | | This work |  |
| YSF18 | | MATa/α *ibid.* pSF37, pSF43 | | | | | | This work |  |
| YSF19 | | MATa/α *ibid.* pSF39, pSF43 | | | | | | This work |  |
| YSF20 | | MATa/α *ibid.* pSF41, pSF43 | | | | | | This work |  |
| YSF21 | | MATa/α *ibid.* pSF39, pSF44 | | | | | | This work |  |
| YSF22 | | MATa/α *ibid.* pSF40, pSF42 | | | | | | This work |  |
| YSF23 | | MATa/α *ibid.* pSF41, pSF42 | | | | | | This work |  |
| YSF24 | | MATa/α *ibid.* pSF40, pSF44 | | | | | | This work |  |
| YSF25 | | MATa/α *ibid.* pSF39, pSF36 | | | | | | This work |  |
| YSF26 | | MATa/α *ibid.* pSF39, pSF38 | | | | | | This work |  |
| YSF27 | | MATa/α *ibid.* pSF41, pSF36 | | | | | | This work |  |
| YSF28 | | MATa/α *ibid.* pSF41, pSF38 | | | | | | This work |  |
| YSF29 | | MATa/α *ibid.* pSF40, pSF36 | | | | | | This work |  |
| YSF30 | | MATa/α *ibid.* pSF40, pSF38 | | | | | | This work |  |
| YSF31 | | MATa/α *ibid.* pSF30, pSF42 | | | | | | This work |  |
| YSF32 | | MATa/α *ibid.* pSF30, pSF44 | | | | | | This work |  |
| YSF33 | | MATa/α *ibid.* pSF30, pSF43 | | | | | | This work |  |
| PCR reactions | | | | | | | | | |
| PCR # | Template/Target | | | Primer 1 (forward) | | | Primer 2 (reverse) | | |
| 55 | mcherry | | | ATGGTGAGCAAGGGCGAGGAGG | | | TTACTTGTACAGCTCGTCCATGCCGCCGG | | |
| 91 | masK | | | GCTCAGGTCGGCGCGCCGATGG | | | CGCACTACCACTAGTCTGCGTCACCCGCCACGAGAAAGG | | |
| 92 | mcherry | | | GGTGAAGCTGCAGCCATGGTGAGCAAGGGCGAGGAGGATAAC | | | TTACTTGTACAGCTCGTCCATGCCGCCGG | | |
| 95 | masK-mcherry  (overlap) | | | GCTCAGGTCGGCGCGCCGATGG | | | TTACTTGTACAGCTCGTCCATGCCGCCGG | | |
| 100 | pUT *phoA* | | | ACGCGTTACCTGTTCTGGAAAACCGGGCTGCT | | | ACGCGTTTATTTCAGCCCCAGAGCGGCTTTCAT | | |
| 101 | pUT *phoA* | | | CTGCAGCCTGTTCTGGAAAACCGGGCTGCT | | | CTGCAGTTATTTCAGCCCCAGAGCGGCTTTCAT | | |
| 102 | WT / *mglBA* | | | GGATCCCGGTACGACCTCAGTGGGC | | | GGATCCGAAGCAACGCGGGAAAAGCG | | |
| 103 | WT/ *masABK* | | | CGTGGAACTGGTTGGGGAACTCG | | | CGACCTGGAGTTCGCCGACCAGG | | |
| 104 | WT/*masB* | | | ATTCCCCGGGGGATCCGTGAGTGCCCCGCGTTGTTGG | | | TACGATTCATAGATCTCGGCCCTCGCTGTTTCAGAGTGCGC | | |
| 105 | WT/p*pilA* | | | TCGCGACGCAGGTGAAGC | | | TCCACTGAAGGAATGCGAGTTACTGG | | |
| 106 | WT/*masA_peri_* | | | ATTCCCCGGGGGATCCCAGCGGGAGGCCTTCG | | | CGACATCGATGGATCCGGCGTCGCGCACCGACAGC | | |
| 107 | WT/*masA_cyto_* | | | ATTCCCCGGGGGATCCCGGCGAGCGGAGCGCTGGACG | | | CGACATCGATGGATCCCCTCTTCCCGAGGGGGAAACGG | | |
| 108 | WT/*pilA*_19-221_ | | | ATTCCCCGGGGGATCCATGATCGTGGTCGCCATCATCG | | | CGACATCGATGGATCCGCAGGCGACGTCGTTGCG | | |
| 109 | WT/*pilR* | | | ATTCCCCGGGGGATCCCTG GGG TCG CGC GGC CAC ATC C | | | CGACATCGATGGATCCCGCATCCGAAGCGCTCCCGGGC | | |
| 110 | WT/*pilS* | | | ATTCCCCGGGGGATCCGTGCGCCCGTCGGAGAGGGG | | | CGACATCGATGGATCCCAGCGGCAACCCCACCACGAACTCG | | |
| 111 | WT/*masA* | | | GGTACCGGAGAGCTTCATGGCGCGCCTGGCGCTGGTGC | | | AAGCTTTCACCGTTTCCCCCTCGGGAAGAGG | | |
| 112 | WT/*pilA* | | | TCGCGACGCAGGTGAAGC | | | TCCACTGAAGGAATGCGAGTTACTGG | | |
| 113 | Δ*mas*/*pilA* | | | TCGCGACGCAGGTGAAGC | | | TCCACTGAAGGAATGCGAGTTACTGG | | |
| RT-PCR targets and primers | | | | | | | | | |
| Target | | | Forward | | | reverse | | |  |
| *16S*rRNA | | | ACGGTCCAGACTCCTACGGG | | | AAGCCGTTGGATGTTAGCCA | | |  |
| *mglA* | | | AGGTCTTCTACGACGCCAGC | | | GTTCAGGTCGTAGCCCTGCTC | | |  |
| *masB* | | | GGCTGGGCGCAATCG | | | GCCGTACCGTCACCGGAA | | |  |
| *sglK* | | | ACTTCGACCAGCGCCTCAT | | | AGGTCCAGCCCGTTGTTG | | |  |
| *pilA* | | | CACCGCGCAGAAGTCGTT | | | CGTTGGCGAAGTCGGAGTA | | |  |
| *pilS* | | | GAGGCGAGAATCCGCATCT | | | GTGAAGAACGGCTCGAACAAG | | |  |
| *pilT* | | | ACGGGTTCGGGCAAGTC | | | ACGCTCGGTGTTGATCTTGTC | | |  |
| *fibA* | | | TCGCTCGACCACTACCACAA | | | AGGTAGAACGCCAGGTTGGA | | |  |
| *epsH* | | | TCCGTGACGTCTACAAGCTGAT | | | ATGGCGGACGCGTTCTC | | |  |
| *difA* | | | GTTGGCCGGCGACAAG | | | CGTCGACGATCTTCGAGATTT | | |  |
| *difG* | | | GGTGGCTGGGCATCCA | | | CCCGTTCGGTCTGATGAATC | | |  |
| *frzS* | | | CGACGCGGCCAACAAG | | | GATTTCCTGGTCCTTTTGATTCA | | |  |

1. Yang R, Bartle S, Otto R, Stassinopoulos A, Rogers M, Plamann L, Hartzell P: AglZ is a filament-forming coiled-coil protein required for adventurous gliding motility of *Myxococcus xanthus*. *J Bacteriol* 2004, 186(18):6168-6178.

2. Julien B, Kaiser AD, Garza A: Spatial control of cell differentiation in *Myxococcus xanthus*. *Proceedings of the National Academy of Sciences of the United States of America* 2000, 97(16):9098-9103.

3. Thomasson B, Link J, Stassinopoulos AG, Burke N, Plamann L, Hartzell PL: MglA, a small GTPase, interacts with a tyrosine kinase to control type IV pili-mediated motility and development of *Myxococcus xanthus*. *Mol Microbiol* 2002, 46(5):1399-1413.

4. James P, Halladay J, Craig EA: Genomic Libraries and a Host Strain Designed for Highly Efficient Two-Hybrid Selection in Yeast. *Genetics* 1996, 144(4):1425-1436.

5. Manoil C, Beckwith J: TnphoA: a transposon probe for protein export signals. *Proceedings of the National Academy of Sciences* 1985, 82(23):8129-8133.

6. Hartzell P, Kaiser D: Function of MglA, a 22-kilodalton protein essential for gliding in *Myxococcus xanthus*. *J Bacteriol* 1991, 173(23):7615-7624.
